# Species Diversity and Distribution Patterns of the Ants of Amazonian Ecuador

**DOI:** 10.1371/journal.pone.0013146

**Published:** 2010-10-01

**Authors:** Kari T. Ryder Wilkie, Amy L. Mertl, James F. A. Traniello

**Affiliations:** Department of Biology, Boston University, Boston, Massachusetts, United States of America; Field Museum of Natural History, United States of America

## Abstract

Ants are among the most diverse, abundant and ecologically significant organisms on earth. Although their species richness appears to be greatest in the New World tropics, global patterns of ant diversity and distribution are not well understood. We comprehensively surveyed ant diversity in a lowland primary rainforest in Western Amazonia, Ecuador using canopy fogging, pitfall traps, baits, hand collecting, mini-Winkler devices and subterranean probes to sample ants. A total of 489 ant species comprising 64 genera in nine subfamilies were identified from samples collected in only 0.16 square kilometers. The most species-rich genera were *Camponotus*, *Pheidole*, *Pseudomyrmex*, *Pachycondyla*, *Brachymyrmex*, and *Crematogaster*. *Camponotus* and *Pseudomyrmex* were most diverse in the canopy, while *Pheidole* was most diverse on the ground. The three most abundant ground-dwelling ant genera were *Pheidole*, *Solenopsis* and *Pyramica*. *Crematogaster carinata* was the most abundant ant species in the canopy; *Wasmannia auropunctata* was most abundant on the ground, and the army ant *Labidus coecus* was the most abundant subterranean species. Ant species composition among strata was significantly different: 80% of species were found in only one stratum, 17% in two strata, and 3% in all three strata. Elevation and the number of logs and twigs available as nest sites were significant predictors of ground-dwelling ant species richness. Canopy species richness was not correlated with any ecological variable measured. Subterranean species richness was negatively correlated with depth in the soil. When ant species were categorized using a functional group matrix based on diet, nest-site preference and foraging ecology, the greatest diversity was found in Omnivorous Canopy Nesters. Our study indicates ant species richness is exceptionally high at Tiputini. We project 647–736 ant species in this global hotspot of biodiversity. Considering the relatively small area surveyed, this region of western Amazonia appears to support the most diverse ant fauna yet recorded.

## Introduction

Despite their abundance [1], species richness [2] and ecological dominance [1], [3]–[8], tropical ants have rarely been the focus of intensive biotic inventories and global patterns of ant diversity, including those of New World tropical forests where ants appear to be especially prominent, are poorly described. Recent studies have shown that Yasuni National Park, which is located directly adjacent to the Tiputini Biodiversity Station (TBS) where we conducted our survey, may be the most diverse region on earth, with apparent world richness records for amphibians, reptiles, bats, and trees and insects projected to be represented by at least 100,000 species per hectare [9]. Although no comprehensive inventory of any insect taxa has been published for this region, a few small-scale studies have been carried out. For example, our previous research on subterranean ant diversity at the TBS in Amazonian Ecuador recorded 47 species [10] and found that ant diversity and species accumulation rates decreased with increasing depth in the soil. The species assemblage of ants collected 12.5 cm below the surface was significantly different from those found at 25, 37.5, and 50 cm, suggesting stratified species distribution below ground. Another recent, small-scale comparative study of ant diversity in primary and secondary forest at TBS identified 101 species [11], while other recent surveys identified 77 species of twig- and litter-nesting ants, as well as 56 species in the genus *Pheidole* [12], [13]

Because of their importance in community dynamics and their ecosystem significance, a better understanding of the patterns of ant diversity would greatly enhance our knowledge of the biogeography, organization and dynamics of tropical communities as well as how their biodiversity would best be conserved.

Here we describe the results of collections made to comprehensively inventory ant diversity at TBS and determine patterns of species distribution. We sampled ants ranging in distribution from the canopy to 50 cm below ground, using fogging, surface baiting, pitfall traps, hand collecting, mini-Winkler devices, and subterranean baiting. We compared species composition among strata to describe how ant diversity and abundance are associated with environmental gradients. To examine ecological correlates of ant distribution patterns, we created a functional group matrix based on diet and nesting habits and thus provide a useful framework to describe and analyze ant community structure.

## Results

### Ant diversity and abundance

We identified a total of 489 ant species (475 not including reproductives) comprising 64 genera in nine subfamilies from 8601 species occurrences in 3 strata (7740 not including reproductives; Tables S1, S2, S3). The most species-rich genera were *Camponotus* (46 species), *Pheidole* (45), *Pseudomyrmex* (30), *Pachycondyla* (25), *Brachymyrmex* (20), and *Crematogaster* (19). The five most abundant genera were Pheidole, Camponotus, Crematogaster, Solenopsis, and Pachycondyla. The seven most abundant species overall were *Crematogaster carinata* (occurring in 271 samples (69%)), *Camponotus femoratus* (161 samples (41%)), *Wasmannia auropunctata* (147 samples (38%)), *Solenopsis SC-06* (134 samples (34%)), *Megalomyrmex foreli* (120 samples (31%)), *Nylanderia cf. steinheili* (116 samples (30%)), and *Cr. brasiliensis* (115 samples (29%)).

**Figure 1:**
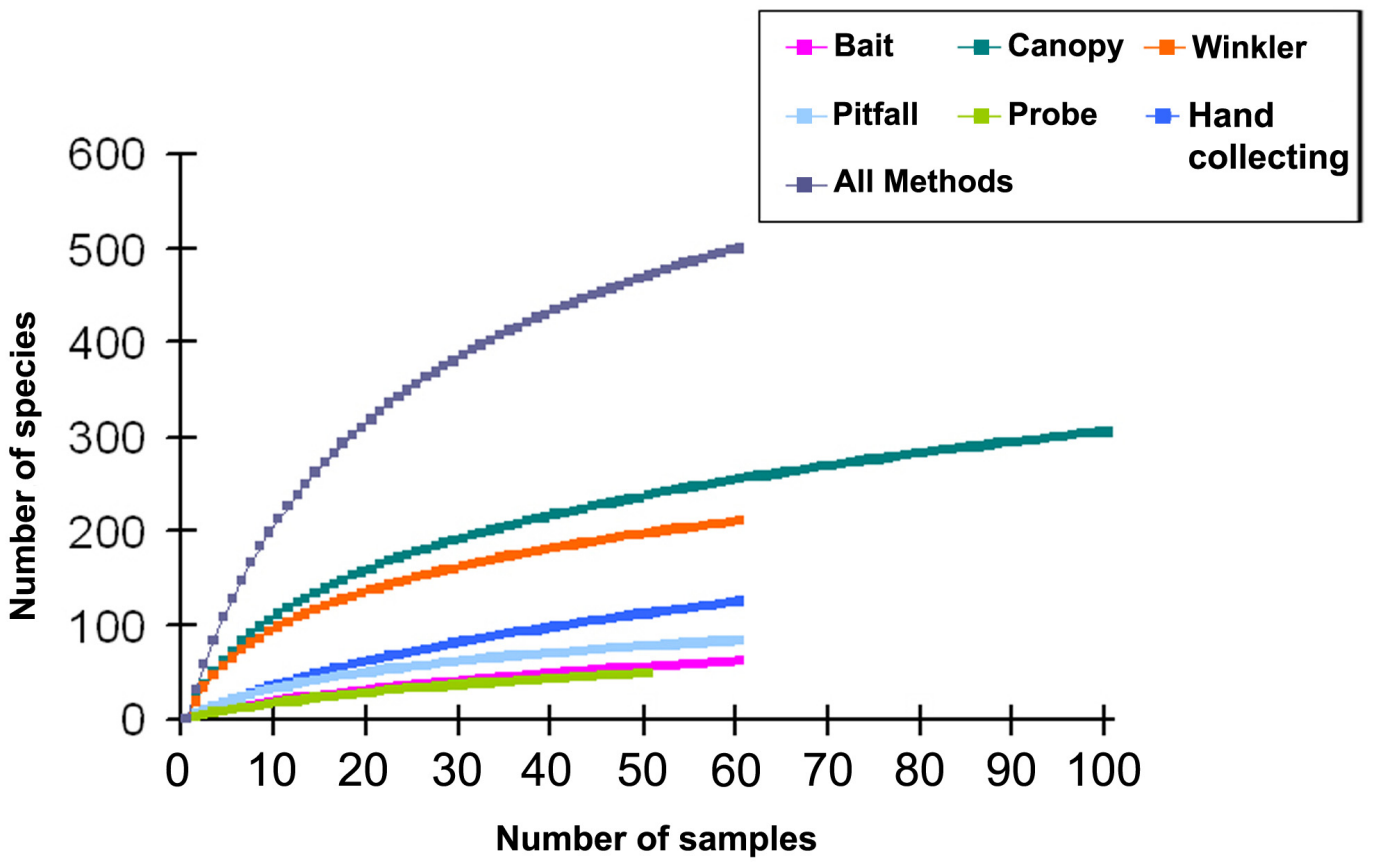
Comparison of the rates of species accumulation for each of six collection methods used. Mao Tau method, 100 replicates; EstimateS 8.0, Colwell 2005.

Species accumulation curves (Figure 1) indicate that canopy fogging collected species at the highest rate, while the subterranean probe had the lowest. Accumulation curves and estimators (Table 1) indicate that additional sampling is required to inventory total species richness at TBS, in spite of our use of six different methods over three strata. Of the three strata, the ground stratum appears to have been the most well-sampled (between 68 and 78% of species sampled), followed by the canopy (62–70%) and subterranean (54–66%) stratum. Nonparametric estimators ICE, Jackknife1, Jackknife2, and Chao2 suggest the actual diversity of ants at TBS is between 647 and 736 species, suggesting we collected approximately 66–76% of species in our survey.

**Table 1:** Estimated proportion of the potential species richness sampled by each collection method within each of the three strata.

Collection Method/Strata	Estimated richness	Sampled richness	Estimated proportion of potential species sampled
Winkler	282–321	185	58–66%
Pitfall	115–134	96	64–75%
Baiting	94–125	83	66–88%
Hand-collecting	192–247	150	61–78%
**Ground strata:**	**347–395**	**269**	**68–78%**
Canopy fogging	405–457	282	62–70%
**Canopy strata:**	**405–457**	**282**	**62–70%**
Probes	73–89	48	54–66%
**Subterranean strata:**	**73–89**	**48**	**54–66%**
*Overall:*	*647–736*	*489*	*66–76%*

### Vertical stratification of ant species

A total of 282 species were identified from canopy fogging samples, 275 from ground samples (83 from baiting, 150 from hand collecting, 96 from pitfall traps, and 185 from Mini-Winklers) and 48 species were collected below the soil surface to a depth of 50 cm using a baited subterranean probe [10]. Across all samples, 80% of species were found in only one stratum (205 in the canopy, 180 on the ground and eight in subterranean samples), 17% in two strata (a total of 62 species in the canopy and on the ground, a total of 18 species on the ground and in subterranean samples, and only one species total in canopy and subterranean samples) and 3% in all three strata.

**Figure 2:**
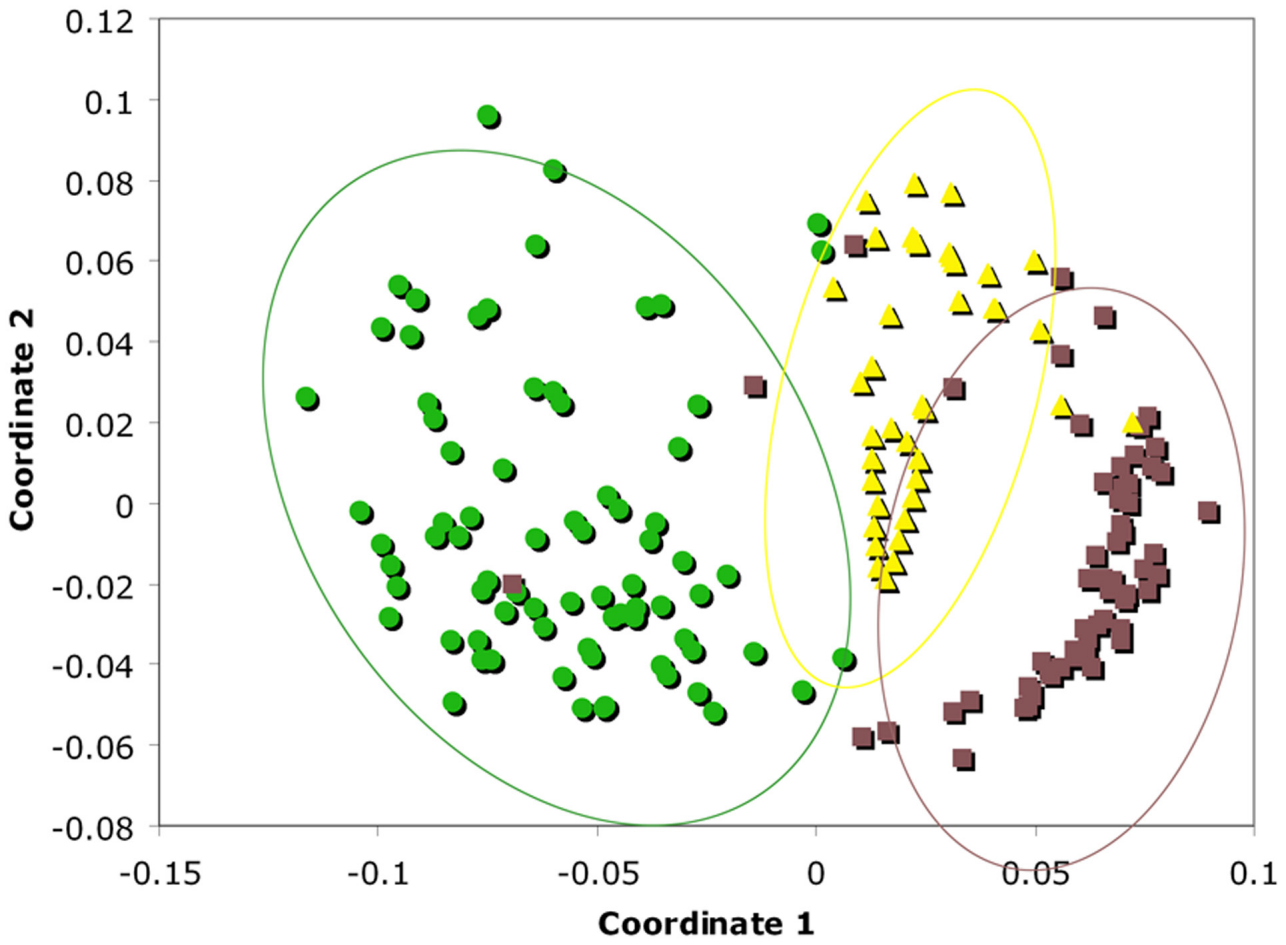
Non-metric multidimensional scaling (Raup-Crick distance measure) differentiating canopy (green), ground (brown), and subterranean (yellow) species. Each symbol represents a single collection sample. Rare species (singletons) were removed prior to analysis, leaving 194 species remaining.

**Figure 3:**
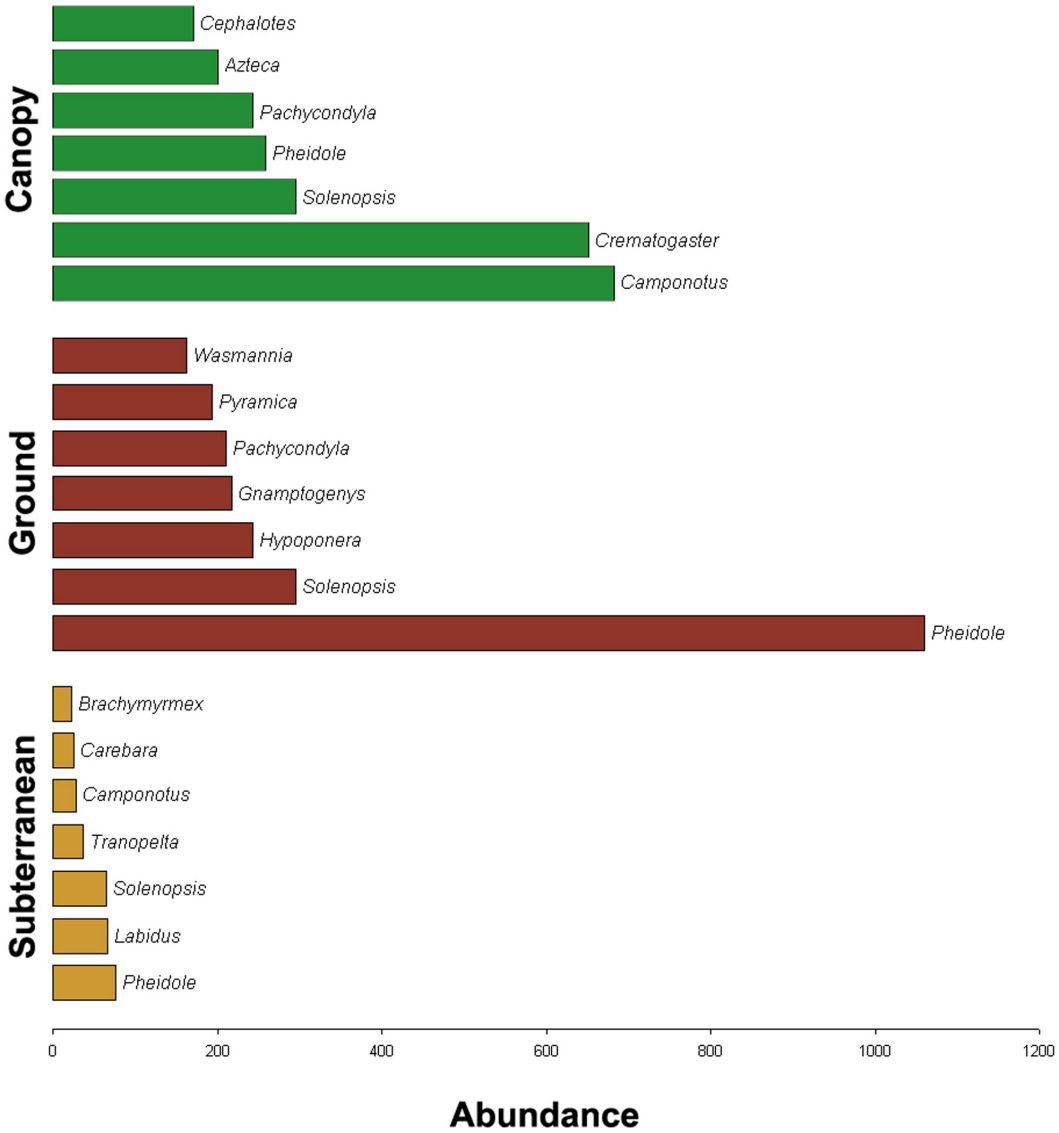
Abundance of individuals by genus. Top 7 genera in each stratum showed, in terms of abundance ( = number of occurrences).

**Figure 4:**
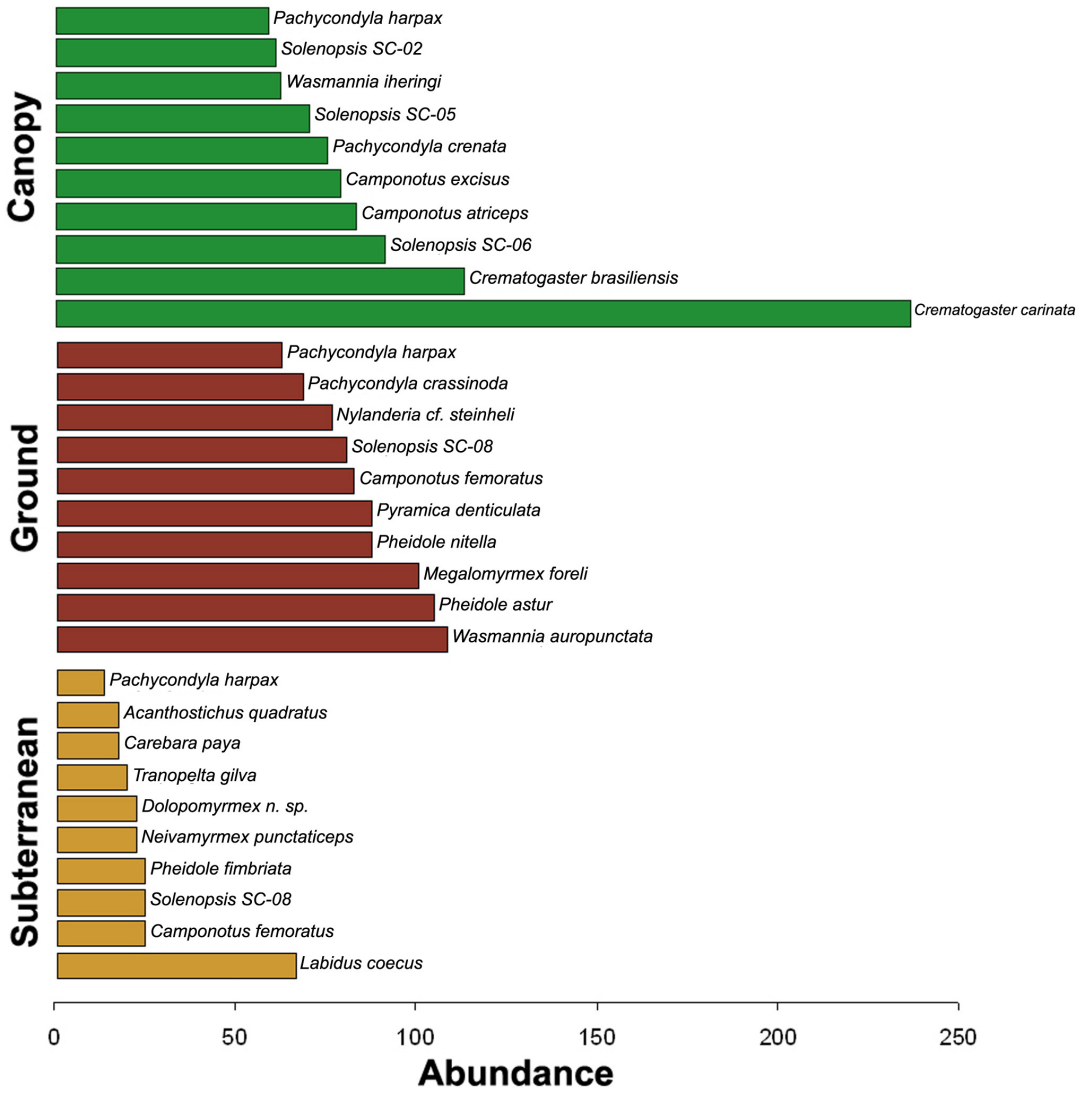
Abundance of individuals by species. Top 10 species in each stratum showed, in terms of abundance ( = number of occurrences).

ANOSIM comparisons indicated highly significant (p≤0.0001) differences among strata in the distribution of ant genera and species diversity, and NMDS analysis showed clear separation between the three strata (Figure 2). The most abundant genera in the canopy were *Crematogaster* and *Camponotus*, while *Pheidole* was the most abundant genus on the soil surface and beneath it (Figure 3). The most abundant ant species in the canopy was *Cr. carinata*; *Wasmannia auropunctata* was most abundant on the ground, and *Labidus coecus*, an army ant, was most abundant in subterranean collections (Figure 4).

### Environmental Correlates of Diversity

**Figure 5:**
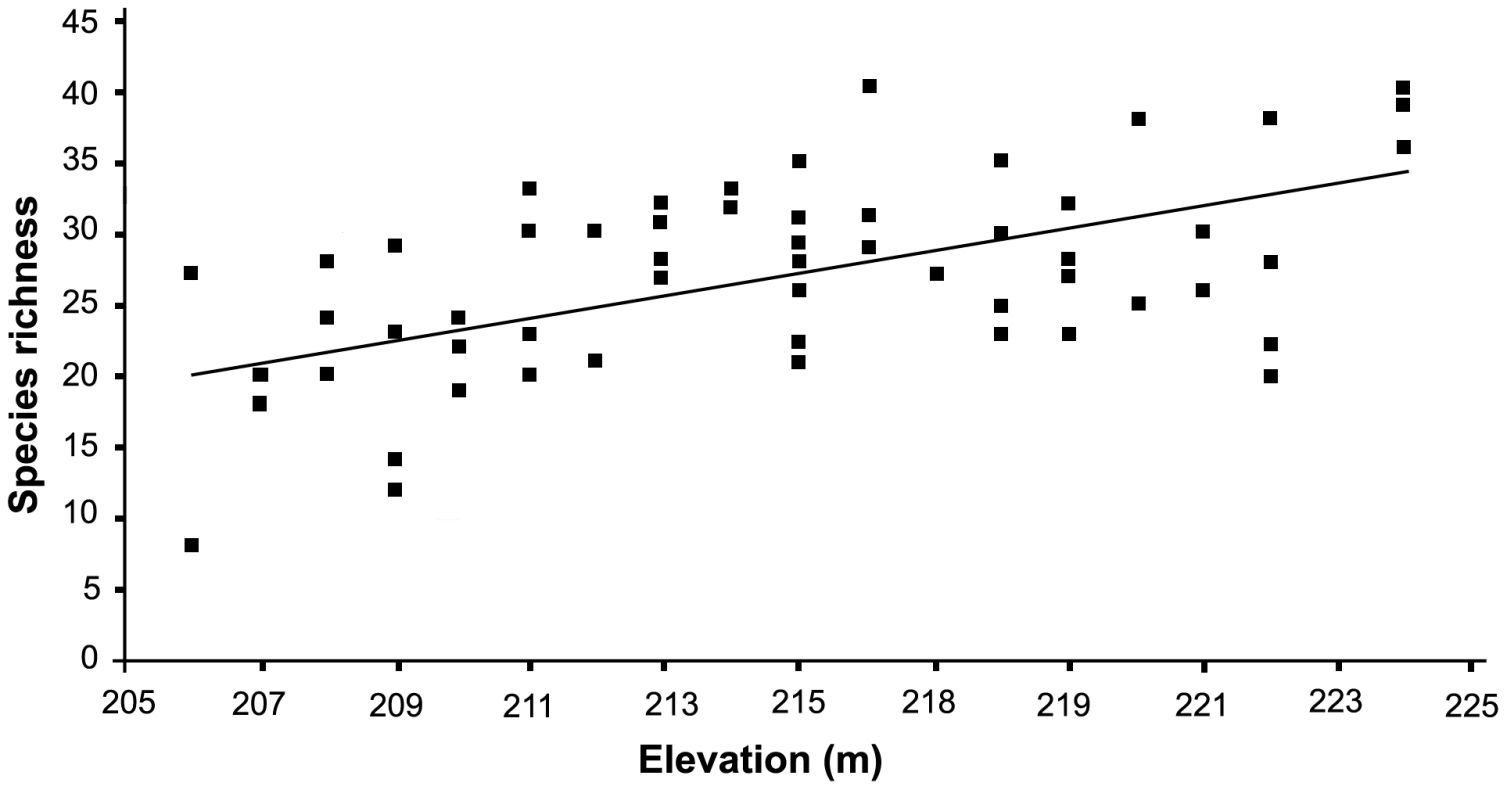
Effect of elevation on ground-dwelling ant species richness. R^2^ = 0.56, n = 60, p<0.0001.

**Figure 6:**
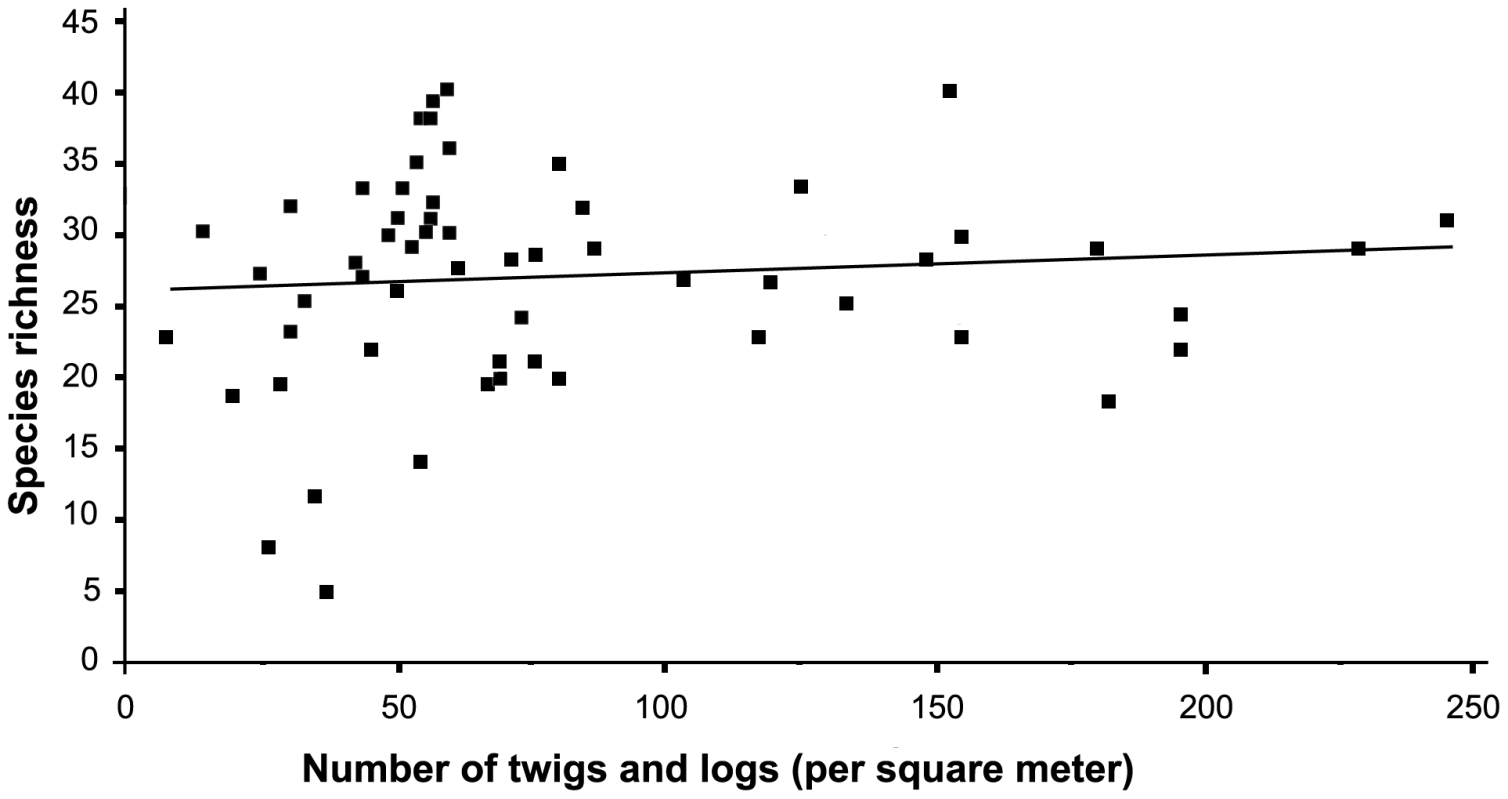
Effect of number of twigs and logs on ground-dwelling ant species richness. R^2^ = 0.27, n = 60, p = 0.035.

The relationship between species richness and environmental variables traditionally correlated with diversity was determined using a linear multiple regression model, which provided a significant fit for ant species richness (*F*_9,50_ = 5.46, *p*<0.0001, adjusted *R^2^* = 0.40). Elevation was a significant predictor of ground-dwelling ant species richness (*t* = 5.83, *p*<0.0001; Figure 5), as was the number of logs and twigs/m^2^ in sample plots (*t* = 4.70, *p* = 0.035; Figure 6). Neither elevation (*R^2^* = −0.039, *p* = 0.951), nor the number of twigs and logs (*R^2^* = −0.003, *p* = 0.56) showed significant spatial dependency, The remaining seven variables (slope, litter depth, vertical height profile, canopy cover, bare ground, number of plants, volume of twigs and logs) were not significant predictors of richness across all species (Table 2). Slope and number of plants were significant predictors of *Pheidole* richness and canopy cover was a significant predictor of *Solenopsis* richness. Elevation and slope were significant predictors of *Pyramica* richness (Table 3). All of these significant associations showed positive correlations. The nine measured environmental variables had no significant relationship to the species richness of ants in the canopy (*F*_9, 20_ = 1.91, *p* = 0.109, adjusted *R^2^* = 0.22) or in subterranean probes (*F*_9, 20_ = 0.429, *p* = 0.904, adjusted *R^2^* = −0.22).

**Table 2:** Environmental variation and ant species richness.

Variable	Mean (SD)	Range	*p*
Elevation (m)	214.7 (5.0)	206–224	**<0.0001**
Slope (°)	12.2 (7.4)	2–39	0.061
Litter depth (cm)	5.9 (1.9)	1.4–11.1	0.373
Vertical height profile	0.5 (0.2)	0.1–0.9	0.097
Canopy cover (%)	92.1 (2.8)	85.2–95.6	0.376
Bare ground (%)	3.5 (11.4)	0–55	0.977
Number of plants/m^2^	13.1 (9.4)	0–39	0.513
Number of twigs and logs/m^2^	87.6 (56)	8–244	**0.035**
Volume of twigs and logs/m^2^ (cm^3^)	3,508.2 (12,070)	33–90,919	0.598

Mean (standard deviation) and range for 60 transect sample sites are shown. p values reflect the significance of each variable as a predictor of ground-dwelling ant species richness. Analysis by a linear multiple regression model. Significant predictors are shown in bold type.

**Table 3:** Environmental variation and species richness for the three most abundant ground-dwelling ant genera.

Variable	*Pheidole*	*Solenopsis*	*Pyramica*
Elevation (m)	0.477	0.074	**0.027**
Slope (°)	**0.012**	0.651	**0.013**
Litter depth (cm)	0.726	0.473	0.838
Vertical height profile	0.743	0.734	0.130
Canopy cover (%)	0.074	**0.040**	0.915
Bare ground (%)	0.586	0.724	0.659
Number of plants/m^2^	**0.020**	0.620	0.533
Number of twigs and logs/m^2^	0.507	0.287	0.205
Volume of twigs and logs/m^2^ (cm^3^)	0.154	0.598	0.567

p values reflect the significance of each variable as a predictor of species richness for each genus. Significant predictors shown in bold type.

### Functional group distribution

Species of some genera (*Pachycondyla*, *Camponotus*, and *Pheidole*) were distributed among several nest-type groups, while all species in other genera (*Tapinoma*, *Nesomyrmex*, and *Hylomyrma*) were characterized by a single nesting habit. In the latter case, this grouping was often due to inferences made about generic similarities when detailed information on the nesting habits of individual species in those genera was lacking, as niche conservatism in nesting site is assumed for less diverse ant genera (i.e. [14]).

**Figure 7:**
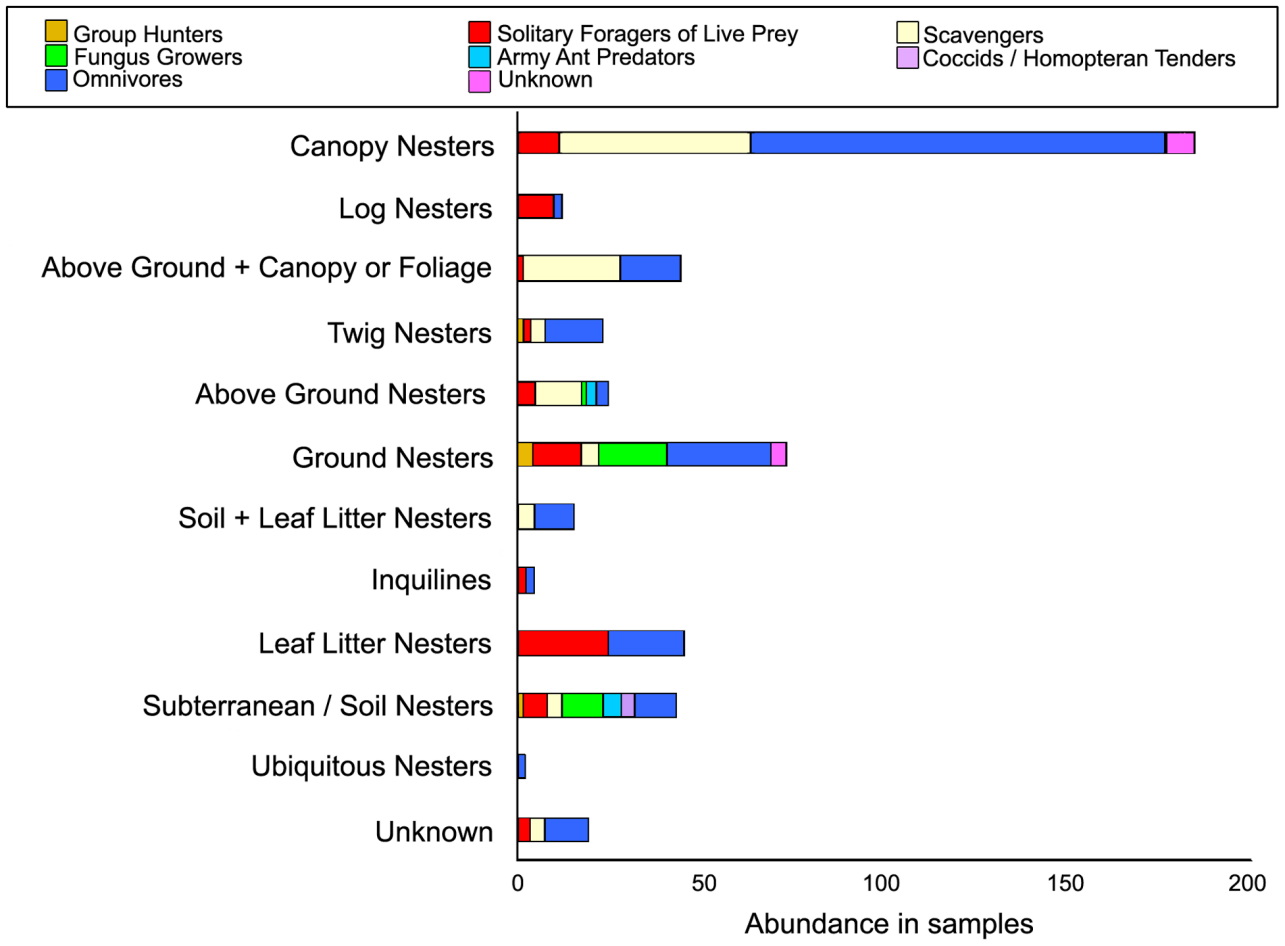
Functional group distribution.

The greatest number of species (113, 23.1%) occurred in the Canopy Nesters/Omnivores category, followed by Canopy Nesters/Scavengers (52, 10.6%), Ground Nesters/Omnivores (28, 5.7%), and Above Ground+Canopy or Foliage Nesters/Scavengers (27, 5.5%; Figure 7).

## Discussion

### Ant diversity and abundance at TBS

Our study of ant species richness in primary forest at TBS represents the first inventory of ants in all above-ground strata and beneath the soil surface in lowland rainforest in Ecuadorian western Amazonia. Of the most species-rich genera, *Camponotus* (41 species) and *Pseudomyrmex* (30 species) were most diverse in canopy samples. *Pheidole* was most diverse on the ground (37 species), and *Solenopsis* (10 species) and *Pheidole* (7 species) were the most diverse subterranean genera. Eighteen genera were represented in our collection by only one species, and three genera by a single specimen each. Species accumulation curves and estimators indicate that actual species diversity of this tropical rainforest hotspot is far greater than the 489 ant species identified in our study, in spite of our intensive and diverse collection methods. We estimate actual diversity at 647–736 species.

Surveys of the ant faunas of other Neotropical regions include Cuzco-Amazonico, Peru – 365 species [15], Urubamba River Valley, Peru – 124 species [16], Panguana, Peru – 520 species [17], Brazilian Amazon – 156 species [14]; 143 species [18], Brazilian Atlantic forest – 124 species [19], tropical Brazilian forests – 206 species [20]; 74 species [21], secondary growth Brazilian tropical forest – 124 species [22], and lowland rainforest in Costa Rica – 437 species [23]. At Yasuni, adjacent to TBS, a pilot study of ant species richness found 109 species (M. Kaspari, personal communication). Worldwide, the only other studies of comparable breadth were carried out in Borneo – 524 morphospecies [24] and Madagascar – 381 species [25]. These studies vary widely in purpose, effort, collection methods, area covered, and identification levels, rendering meaningful comparisons difficult. Majer et al. [26] sampled ants using unit-time hand collecting (day and night), sweeping, beating, baiting and Winkler sacks to compare the ant communities of annually inundated and *terra firme* forests. In contrast, Silva et al. [19] used only Mini-Winklers and baits to examine ant diversity along a habitat regeneration gradient in the southern Brazilian Atlantic Forest. Using a broad array of methods to sample across strata, our study identified 489 ant species within only 16 hectares, an area less than 2% the size of other studies with comparable richness. For example, Verhaagh et al. [17] found 520 species in 1000 hectares surveyed and Longino et al. [23] collected 437 species in 1500 hectares. Although direct comparisons of species richness between sites can not be definitive, it is nevertheless clear that the ant fauna of TBS is among the most diverse yet recorded.

Our results agree with previous studies [27] that identified *Pheidole*, *Camponotus*, and *Crematogaster* as the most prevalent ant genera globally. Ant abundance, however, differed according to strata in our study. In the canopy, *Camponotus* and *Crematogaster* were the most abundant genera, occurring in 35% of all canopy samples, *Cr. carinata* and *Cr. brasiliensis* comprising 12% of the total. The most abundant *Camponotus* species in the canopy were *C. atriceps* and *C. excisus*. The genus *Pheidole* was overwhelming on the ground, comprising 26% of all ground samples. *Wasmannia auropunctata*, *Pheidole astur*, and *Megalomyrmex foreli* were the most abundant species on the ground, although each occurred in only ∼3% of all ground samples. *Pheidole* was most abundant below ground as well, occurring in 18% of all subterranean samples, although *Labidus coecus* was the most abundant species found below-ground (16% of all subterranean samples). We found more species of *Camponotus* than *Pheidole* overall, but the species richness of *Pheidole* was potentially under-sampled, as many minor workers could not be definitively identified to species or morphospecies because major workers were not collected. In fact, 67 species of *Pheidole* have been collected at TBS [12].

### Vertical stratification of ant species

Vertical stratification of ants in canopy, ground, and subterranean habitats was striking: 80% of species were found in only one stratum and only 3% of species were found in all three strata. Non-metric multidimensional scaling showed a clear differentiation of ant species among strata. The vertical distribution of the most abundant species showed that while some species were found predominantly in one strata (i.e. *Crematogaster carinata* in the canopy and *Megalomyrmex foreli* on the ground), other species (i.e. *Camponotus femoratus*) were more evenly distributed among the three strata. The three most common genera (*Pheidole*, *Camponotus*, and *Crematogaster*) clearly showed vertical partitioning, with *Camponotus* and *Crematogaster* dominating in the canopy, and *Pheidole* dominant on the ground. The relatively large body size and number of *Camponotus*, in addition to its aggressiveness and territoriality may contribute to their dominance in the canopy. Recent work has also focused on the relationship between members of the tribe Camponotini (which includes *Camponotus*) and their endosymbiotic *Blochmannia* bacteria, which may have allowed them to have a nutritionally unbalanced diet (honeydew) unavailable to other ants [28]. *Crematogaster*, although smaller in size and less aggressive, may benefit from their parabiotic association with *Camponotus* (seen most frequently between members of the *Crematogaster limata* complex, including *Cr. carinata* and *Cr. brasiliensis*, and among *Camponotus*, most often *Ca. femoratus*) [29]–[32]. Conversely, small body size and colony size in *Pheidole* may have been an important adaptation for nesting in the leaf litter, contributing to their ground-dwelling dominance and diversification [33].

Our results agree with those of prior studies describing strong vertical stratification in tropical ant communities [24], [34]–[36]. Similar patterns have been found among other Neotropical taxa, including collembolans [37], termites [38], birds [39], bats [40] and other small mammals [41]. Multiple hypotheses have been suggested to explain this pattern [42]. The determinants of most arthropod stratification tend to include abiotic factors, forest structure, resource availability, and species-typical behavior [43]. We do not have sufficient data to fully examine the causes of stratification in the ant fauna of TBS; however, we did find evidence of differing effects of environmental factors and variation in diet and foraging ecology among species occupying different strata, as discussed below.

### Environmental Correlates of Diversity

Despite modest differences (205–225 m), it was surprising that elevation was significantly correlated with the richness of ground-dwelling ants. Some studies have shown elevation is a significant determinant of ant diversity, although most indicate lower diversity at higher elevations [44]–[46] or a peak in diversity at mid-elevations [25], [45], [47]–[49]. However, these studies have generally examined a much wider elevational range (300–1650m [49]; 250–1750m [48], and 785 to 1650m [45]) whereas our study gradient was microtopographic. Soil type and drainage may also be important: small differences in elevation could result in significant differences in the degree of soil drainage and aeration, which may in turn affect the microinvertebrate community that many ants rely upon for food. However, this would predict a decrease in subterranean ant diversity at lower elevations, a hypothesis that was not supported. Variation in the availability of prey species or other food resources could also be associated with elevational changes [49]; perhaps this relationship also occurs at our more narrow scale In this regard, we found a significant correlation between ant diversity and termite diversity at TBS (Ryder Wilkie et al., in prep). Termites are a common, and often preferred, prey of many ant species.

The number of twigs and logs was also significantly correlated with ant species richness. Many ant species, including *Camponotus WM-010*, *Ca. planatus*, *Leptogenys imperatrix*, and *Lachnomyrmex scrobiculatus* in our study, nest exclusively in twigs or logs. At least 77 ant species are known to inhabit twig nests at TBS [13]. The availability of these nest sites could limit ant diversity. Increased twig variation has been show to increase ant diversity [50], but because we did not measure variation in twig availability, further research is required to determine its impact on ground-foraging ant ecology at our study site. Considering the three most abundant ground-dwelling genera separately, significant distributional correlations were found for *Pheidole* (slope and number of plants), *Solenopsis* (canopy cover), and *Pyramica* (elevation and slope). Tropical *Pheidole* are known to interact ecologically with plants [51], [52], perhaps explaining the relationship between their diversity and plant abundance. However, the remaining results are not as intuitively understood. Both *Pheidole* and *Pyramica* contain a high proportion of litter-nesting species, and depth of litter could be affected by slope. The lack of significant correlation between diversity in these genera and either twig abundance or litter depth, however, makes this explanation unlikely. Similarly, degree of canopy cover could affect ground temperature and therefore ant foraging [53], but we do not sufficiently understand the foraging behavior of ground-dwelling genera to predict why only *Solenopsis* showed this pattern. Overall, our results indicate that the diversity and distribution patterns of individual taxa are influenced by a mosaic of factors, each potentially affecting species distribution separately or in combination.

None of the environmental factors we measured had a significant effect on ant diversity in the canopy or below ground. This is not particularly surprising for canopy diversity, as measurements were made on the ground and in the understory, although factors such as elevation and canopy cover could still affect the canopy environment. More surprising is the lack of significant association between elevation and the diversity of the subterranean ant fauna, a result which provides additional support for the strong separation of ground-dwelling and subterranean ant communities and the abiotic factors influencing ant diversity above and below ground.

### Ant Functional Groups at TBS: Diet, Foraging ecology, Nesting Habits and Species Distribution

Several attempts to analyze community dynamics have been made to categorize ants according to diet, nesting habits and competitive interactions. Andersen introduced a functional group classification for the desert ants of Australia, a system later used to compare the Australian and North America faunas [54], South African species [55], and rainforest ants globally [56]. This classification system, however, may have limited utility when applied to Neotropical ants because ant functional groups in South America do not consistently correlate with those of Australia. Fungus-growing ants (Tribe Attini), for example, have no Australian equivalent, yet attine ants appear to play a major role in Amazonian community ecology. In other cases, analogous faunas exist in Australia, but differ in prominence, such as the less prevalent Dominant Dolichoderinae or more dominant Subordinate Camponotini [57]. We therefore created a functional group matrix based on diet, foraging ecology and nesting habit that is more suitable to Neotropical ants. In our survey, more canopy species were found than species in any other habitat, and among dietary preferences, omnivorous species were the most numerous. Omnivorous Canopy Nesters, comprised of the majority of *Azteca* (the only genus of the dominant Dolichoderinae group), *Pseudomyrmex*, *Procryptocerus*, and *Cephalotes*, as well as other species, thus had the highest species diversity of any of our functional groups, Scavenger Canopy Nesters (including many *Camponotus*, *Crematogaster*, and *Dolichoderus*), and Omnivorous Ground Nesters (mostly *Camponotus*; Figure 7) also showed high species diversity compared to other functional groups. Ants are known to be particularly dominant and abundant in the canopy; this may be due to their omnivory, including the ability to scavenge and utilize homopteran and extrafloral nectary secretions [58], [59]. Recent work [60] suggests that bacterial gut symbionts may have influenced the diversification of omnivorous ants in tropical canopies. It is important to note, however, that canopy fogging was the most intensive mode of sampling in our study (100 sites, each repeated 9 times, compared to our combined ground-sampling methodologies of 60 sites, no repetition). Therefore, ants in ground-dwelling functional groups may be underrepresented.

Explanations for the high diversity of ant species in the tropics have often focused on habitat specialization and niche partitioning. Our results support the findings of Tobin [61] and Vasconcelos and Vilhena [35], which suggest that habitat specialization is an important factor in the organization and exceptional diversity of tropical rainforest ant communities. High niche diversity in the Neotropics is thus thought to drive specialization and support high species diversity.

Species in genera for which detailed dietary and nesting information are known were distributed widely within the functional group matrix (for example, *Pachycondyla* species spanned 10 categories). In genera for which little or no information is available, species were placed in one functional group. For example, all species of *Hylomyrma* were categorized as Scavengers/Soil and Leaf Litter Nesters, whereas all species of *Nesomyrmex* were categorized as Unknown Canopy Nesters, but if more detailed dietary and nesting information was known, individual species within these genera might more appropriately be placed into different functional groups. The lack of behavioral and ecological information regarding the majority of Neotropical ant species limits more detailed analysis. Further studies examining dietary preferences in ants [7], [58] will contribute to better delineating patterns of ant distribution, abundance and diversity.

### Conclusion

Our comprehensive survey of the ant fauna at TBS suggests western Amazonia holds the most diverse ant fauna described to date. Our study provides a model for future surveys and comparative analysis of Neotropical ant diversity and abundance and establishes a foundation for continuing research in the Ecuadorian Amazon, a region that has global conservation significance. By sampling the subterranean fauna to capture the full spectrum of ant habitats and ecology, our data represent a broader inventory of ant species diversity and distribution than has hitherto been described. Although more detailed ecological characterization of the diverse fauna of western Amazonia will be required to fully understand the patterns underscoring its remarkable diversity, we were able to identify a number of key factors, including the increase in ground-dwelling ant diversity with increasing elevation and litter abundance, the dominance of *Camponotus* and *Crematogaster* in the canopy and *Pheidole* on the ground, the clear stratification of ant communities between the canopy, ground and subterranean habitats, and the prevalence of omnivorous, canopy nesting ant species. By improving our understanding of the diversity of Neotropical ants, a keystone ecological group, we can advance our knowledge of the causes that maintain biodiversity in exceptionally rich Neotropical habitats.

## Methods

Ants were collected at the TBS in the western Amazonian rainforest of Ecuador (Orellana Province, Ecuador, S 00°37′.55″ and W 076°08′39″, annual rainfall≈3000 mm), bordering Yasuní National Park. The study site comprises a 16 hectare area with an elevational range from 206m to 224m. The study site is predominantly primary lowland rainforest, which has a diverse tree community dominated by the palm *Iriartea deltoidea* [62]. The area has been identified as a major tropical wilderness of exceptional richness and is one of 25 global biodiversity hotspots [9], [63]. All samples other than canopy and subterranean samples were collected between 10 February and 3 March, 2003. Canopy samples were collected by Dr. Terry Erwin between January, 1994 and July, 2002 [64]. Subterranean samples were collected 8–26 August, 2004. A total of ∼113,000 ants were collected, the majority from the canopy.

**Figure 8:**
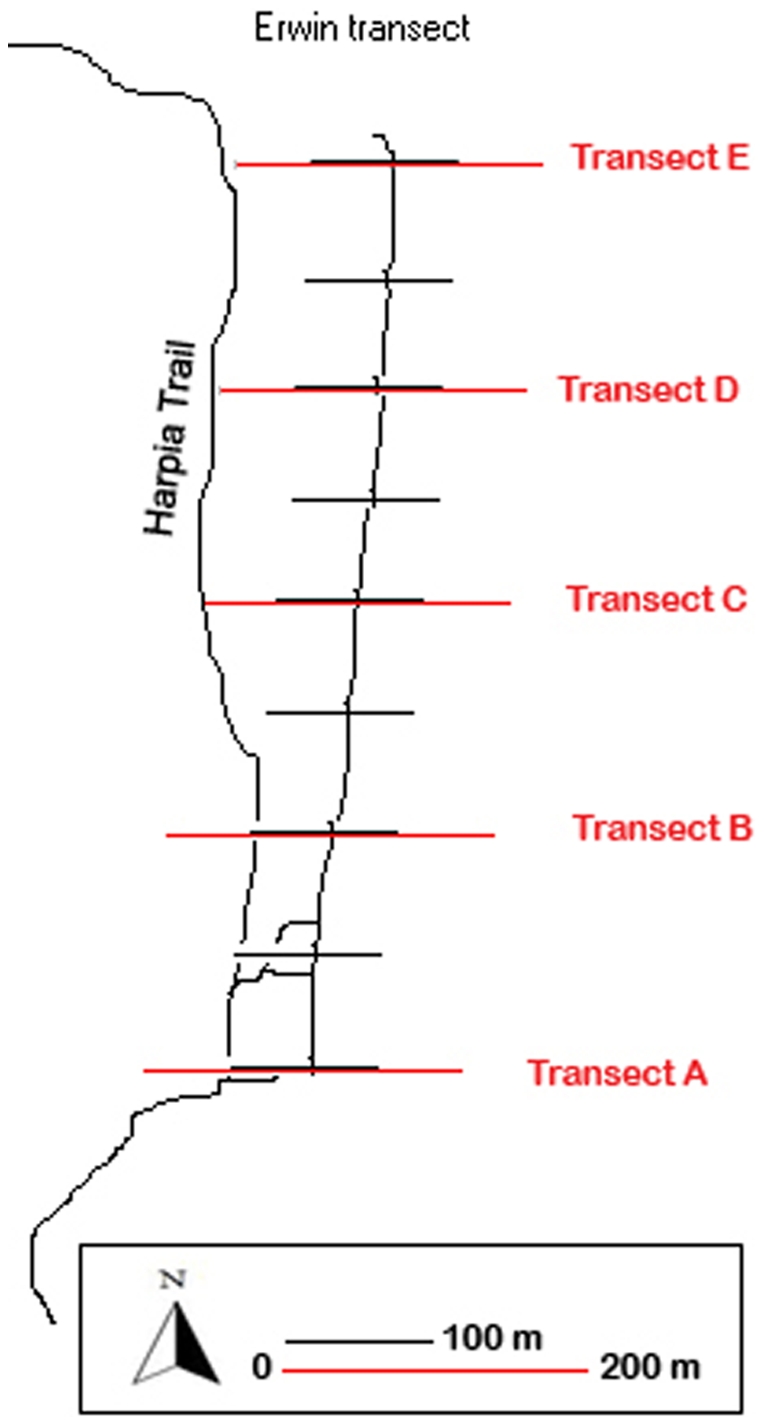
Map of sampling transects. Black lines indicate canopy fogging sample transects of Dr. T. Erwin; red lines indicate transects (ground and subterranean) of present study.

In order to sample ants from our three strata of interest (ground, canopy and subterranean), we used a variation of the Ants of the Leaf Litter (ALL) protocol [65], which is commonly used in studies of tropical ant diversity. Three 200 m transects were established (Transects A, B, and C; Figure 8), each divided into 20 collection sites, each 10 m apart. At each collection site, the following methods were used to sample ground-dwelling ants (ants that nest or forage on or in the leaf litter): 1) A Mini-Winkler device [25] was used to extract ants from one square meter of leaf litter at each collection site. Sacks filled with sifted litter were suspended for 48 hours before ants were removed. 2) Pitfall traps made of plastic containers (diameter = 9 cm, volume = 400 ml) were filled with approximately 130 ml of 96% isopropanol. After 48 hours, contents were collected and stored for future study. 3) Tuna, peanut butter, cookie, and quinoa baits, each roughly 1–2 cm^3^, were set out at each collection site. Baits were placed on a 3.5 mm×3 mm index card, and ants occupying baits were collected after 30 minutes. 4) Hand collecting was accomplished during 15 minutes at each collection site by carefully examining twigs, logs, litter, the soil surface, and tree branches and trunks in a 10 m×2 m area. In addition, ant diversity and abundance in the canopy and underground were sampled using the following methods: 1) Five 200 m transects (the three transects used in ground collection samples (A, B, and C) and two additional transects (D and E; Figure 8) were sampled using a subterranean probe [10] and 2) the canopy was fogged multiple times between 1994 and 2002 with pyrethrin along ten 100 m transects (Transects A through J; see Erwin et al. [64] for details).

To ecologically map patterns of ant distribution, the following environmental gradients were measured at each collection site (instrumentation/methodology noted in parentheses): elevation (altimeter), slope and aspect (clinometer and compass), canopy cover (crown illumination ellipses index) [66]. Leaf litter depth (average of 10 measurements), bare ground percentage, number of plants/m^2^, number of twigs and logs/m^2^, and volume of twigs and logs/m^2^ were also collected in a 1 m^2^ area at each collection site (separate from the 1m^2^ used for Mini-Winklers)

Specimens were identified to species using keys [33], [67]–[78] or were identified by experts (Stefan Cover - *Solenopsis*; Shawn T. Dash - *Hypoponera*; Stephanie Johnson - *Azteca*; John Longino - *Crematogaster*, *Wasmannia*; William Mackay - *Camponotus*, *Pachycondyla*; Ted Schultz – *Apterostigma*, *Cyphomyrmex*, *Mycocepurus*, *Sericomyrmex*, *Trachymyrmex*; Jeffery Sosa-Calvo - *Myrmicocrypta*, *Pyramica*, *Strumigenys*; James Trager – *Nylanderia*). Whenever possible, ants were compared to specimens in the collection of the Harvard Museum of Comparative Zoology (MCZ), where vouchers have been deposited.

EstimateS [79] was used to generate species accumulation curves (Sobs, Mau Tau), which were fit to a logarithmic model. Estimated species richness was calculated using four estimators (ICE, Jackknife1, Jackknife2, and Chao2) recommended by Hortal et al. [80] as the most accurate for our data. For this analysis, all collection methods were combined and total species richness at each site was used as a sample. Analyses of similarity (ANOSIM) [81] comparisons were made using the one-way ANOSIM function in the PAST software package [82] to detect differences in the ant diversity of different strata. ANOSIM was calculated using the Bray-Curtis Similarity Index, a widely used and well-tested index for incidence data [83], [84]. We also evaluated the relative abundance of each ant genera (overall, by sampling method, and within our three strata of interest) based on their number of occurrences in samples. Although such estimators may either underestimate [23] or over estimate actual species richness, particularly in samples with high numbers of rate species [85], they are still useful for comparing between sampling methods and strata.

Multiple regression was used to determine the effect of environmental variation on ground-dwelling ant richness at our 60 principal sites (20 sites each along transects A, B and C). Species richness of ground-dwelling ants was measured for each of the 60 principal sites based on the incidence of species in pitfall traps, baiting stations and Mini-Winkler samples. Richness was regressed against nine environment variables: elevation (m), ground slope (0–90°), leaf litter depth (cm), vertical height profile, canopy cover (%), amount of bare ground (%), number of plants/m^2^, number of twigs and logs/m^2^ and the total volume of twigs and logs/m^2^. An exploratory correlation coefficient matrix of these nine variables showed that all were at most weakly associated (*R^2^*≤0.41), supporting the independence of variables, and enabling multiple regression (least squares method). Slope, number of twigs and logs, and total volume of twigs and logs were log-transformed prior to regression to improve normality. Canopy cover, percent bare ground and number of plants could not be normalized through transformation. However, a linear model provided a good fit to the data based on plots showing no correlation between residuals and fitted values (*F*<0.001, *p*>0.05 for all regressions), and normally distributed residuals. Separate regressions were run for total ant species richness, as well as the richness of the three most abundant ground-dwelling ant genera, *Pheidole, Solenopsis* and *Pyramica*. Species collected in canopy fogging and subterranean probes were not included in measurements of ground-dwelling species richness; however, we also conducted separate multiple regressions for each of these two collection methods. Regression was performed using JMP version 5.0.1 (SAS Institute Inc., Cary, NC, 1989–2002). We used the Mantel test to determine the potential impact of spatial auto-correlation on all significant predictive variables, based on Raup-Crick distance measures. The Mantel test was performed in the PAST software package [82].

Non-metric multidimensional scaling analysis (NMDS) was performed on canopy, ground (pitfall, baiting, Winkler, and hand-collecting combined) and subterranean samples using the PAST software package [82] to visualize differences in distribution patterns among strata based on Raup-Crick distance measures The analysis was run without the inclusion of rare species, to avoid potential bias. “Rare” species were defined as those species found in only one sample, based on visual inspection of a histogram of species abundances.

Information regarding diet and nesting habits were used to create a functional group matrix to reveal patterns of community structure. Species were placed into functional groups based on personal observations of foraging behavior and food choice, nesting ecology, and natural history descriptions taken from available literature. To facilitate ecological comparisons, all ant species collected at TBS were assigned a position in a functional group matrix consisting of seven diet groups (which included elements of foraging behavior) and 11 nest-type groups (Table S4). The seven diet groups were: 1) Group Hunters (cooperatively capture live prey that are large relative to individual worker body size); 2) Solitary Foragers of Live Prey (hunt prey that are small relative to worker body size); 3) Scavengers (collect mostly dead or moribund prey or other food items and infrequently collect live prey); 4) Fungus Growers; 5) Army Ants; 6) Homopteran Tenders; and 7) Omnivores (scavenge for dead prey, capture live prey, collect seeds and plant parts, and visit extrafloral nectaries). If diet could not be determined, species were placed in the category “Unknown.”

The 11 nest-type groups were: 1) Subterranean/Soil Nesters; 2) Leaf -Litter Nesters; 3) Inquilines; 4) Soil- and Leaf-Litter Nesters (i.e., nest in both soil and leaf litter); 5) Ground Nesters (nest in soil, leaf litter, twigs and logs); 6) Above-Ground Nesters (nest in leaf litter, twigs, and logs, but not soil or canopy); 7) Twig Nesters; 8) Above-Ground and Canopy or Foliage Nesters (nest in leaf litter, twigs, logs, and canopy, but not soil); 9) Log Nesters; 10) Canopy Nesters; and 11) Ubiquitous Nesters (species whose nests are found in all habitat groups). Species whose nesting habits have not been described or observed in the present study were categorized as “Unknown.”

## Supporting Information

Table S1 Species list of ants collected at TBS.
(0.32 MB DOC)

Table S2 Summary of results of sampling methods.
(0.03 MB DOC)

Table S3 Abundance (number of occurrences in each sample) by sampling method of each species.
(0.67 MB DOC)

Table S4 Functional group designation of ants at TBS.
(0.09 MB DOC)
