# Correction: Species Diversity and Distribution Patterns of the Ants of Amazonian Ecuador

**DOI:** 10.1371/annotation/832d6104-4f9f-42eb-88a5-b2b1fc4480ca

**Published:** 2010-10-29

**Authors:** Kari T. Ryder Wilkie, Amy L. Mertl, James F. A. Traniello

The third author should be listed under "Conceived and designed the experiments." The correct contribution should read: "Conceived and designed the experiments: KTRW ALM JFAT"

