# Supplementary material for: Species Diversity and Distribution Patterns of the Ants of Amazonian Ecuador
Source: PLoS One. 2010 Oct 1;5(10):e13146. doi: 10.1371/journal.pone.0013146 (PMC2948521; doi:10.1371/journal.pone.0013146)
Supplement: Table S1 — Species list of ants collected at TBS. (0.32 MB DOC) [file pone.0013146.s001.doc]

**Table S1.** Species list of ants collected at TBS.

| *Acanthoponera minor* |
| --- |
| *Acanthoponera peruviana* |
| *Acanthostichus quadratus* |
| *Acromyrmex coronatus* |
| *Acropyga decedens* |
| *Acropyga donisthorpei* |
| *Acropyga fuhrmanni* |
| *Acropyga guianensis* |
| *Amblyopone cf. cleae* |
| *Anochetus bispinosus* |
| *Anochetus diegensis* |
| *Anochetus mayri* |
| *Apterostigma auriculatum* |
| *Apterostigma sp.1* |
| *Apterostigma sp.2* |
| *Apterostigma sp.3* |
| *Apterostigma sp.4* |
| *Apterostigma sp.5* |
| *Apterostigma sp.6* |
| *Apterostigma sp.7* |
| *Azteca SJ-A* |
| *Azteca SJ-AA* |
| *Azteca SJ-B* |
| *Azteca SJ-BB* |
| *Azteca SJ-C* |
| *Azteca SJ-CC* |
| *Azteca SJ-D* |
| *Azteca SJ-DD* |
| *Azteca SJ-E* |
| *Azteca SJ-EE* |
| *Azteca SJ-F* |
| *Azteca SJ-FF* |
| *Azteca SJ-G* |
| *Azteca SJ-GG* |
| *Azteca SJ-H* |
| *Azteca SJ-HH* |
| *Azteca SJ-I* |
| *Azteca SJ-II* |
| *Azteca SJ-J* |
| *Azteca SJ-JJ* |
| *Azteca SJ-KK* |
| *Azteca SJ-LL* |
| *Azteca SJ-M* |
| *Azteca SJ-MM* |
| *Azteca SJ-N* |
| *Azteca SJ-NN* |
| *Azteca SJ-O* |
| *Azteca SJ-OO* |
| *Azteca SJ-P* |
| *Azteca SJ-PP* |
| *Azteca SJ-Q* |
| *Azteca SJ-R* |
| *Basiceros conjugans* |
| *Basiceros manni* |
| *Basiceros militaris* |
| *Brachymyrmex cavernicola* |
| *Brachymyrmex KTRW-001* |
| *Brachymyrmex KTRW-002* |
| *Brachymyrmex KTRW-003* |
| *Brachymyrmex KTRW-004* |
| *Brachymyrmex KTRW-005* |
| *Brachymyrmex KTRW-006* |
| *Brachymyrmex KTRW-007* |
| *Brachymyrmex KTRW-008* |
| *Brachymyrmex KTRW-009* |
| *Brachymyrmex KTRW-010* |
| *Brachymyrmex KTRW-011* |
| *Brachymyrmex KTRW-012* |
| *Brachymyrmex KTRW-013* |
| *Brachymyrmex KTRW-014* |
| *Brachymyrmex KTRW-015* |
| *Brachymyrmex KTRW-016* |
| *Brachymyrmex KTRW-017* |
| *Brachymyrmex KTRW-018* |
| *Brachymyrmex KTRW-019* |
| *Camponotus abscisus* |
| *Camponotus arboreus* |
| *Camponotus atriceps* |
| *Camponotus bidens* |
| *Camponotus bispinosus* |
| *Camponotus bradleyi* |
| *Camponotus branneri* |
| *Camponotus brevis* |
| *Camponotus cacicus* |
| *Camponotus callistus* |
| *Camponotus claviscapus* |
| *Camponotus constructor* |
| *Camponotus emeryodicatus* |
| *Camponotus eurynotus* |
| *Camponotus excisus* |
| *Camponotus femoratus* |
| *Camponotus formiciformis* |
| *Camponotus helleri* |
| *Camponotus hippocrepis* |
| *Camponotus integellus* |
| *Camponotus latangulus* |
| *Camponotus linnaei* |
| *Camponotus macrochaeta* |
| *Camponotus mocsaryi* |
| *Camponotus mus* |
| *Camponotus nidulans* |
| *Camponotus nitidior* |
| *Camponotus novogranadensis* |
| *Camponotus orthocephalus* |
| *Camponotus planatus* |
| *Camponotus rapax* |
| *Camponotus senex* |
| *Camponotus sexguttatus* |
| *Camponotus WM-001* |
| *Camponotus WM-002* |
| *Camponotus WM-003* |
| *Camponotus WM-004* |
| *Camponotus WM-005* |
| *Camponotus WM-006* |
| *Camponotus WM-007* |
| *Camponotus WM-008* |
| *Camponotus WM-009* |
| *Camponotus WM-010* |
| *Camponotus wytsmani* |
| *Carebara angulata* |
| *Carebara panamensis* |
| *Carebara paya* |
| *Carebara urichi* |
| *Carebarella KTRW-001* |
| *Centromyrmex alfaroi* |
| *Cephalotes atratus* |
| *Cephalotes cordatus* |
| *Cephalotes laminatus* |
| *Cephalotes maculatus* |
| *Cephalotes manni* |
| *Cephalotes marginatus* |
| *Cephalotes minutus* |
| *Cephalotes n. sp. near maculatus* |
| *Cephalotes n. sp. near palta* |
| *Cephalotes opacus* |
| *Cephalotes pallidus* |
| *Cephalotes pavonii* |
| *Cephalotes peruviensis* |
| *Cephalotes ramiphilus* |
| *Cephalotes spinosus* |
| *Cephalotes umbraculatus* |
| *Crematogaster acuta* |
| *Crematogaster brasiliensis* |
| *Crematogaster carinata* |
| *Crematogaster crucis* |
| *Crematogaster curvispinosa* |
| *Crematogaster egregior* |
| *Crematogaster erecta* |
| *Crematogaster flavomicrops* |
| *Crematogaster foliocrypta* |
| *Crematogaster JTL-022* |
| *Crematogaster JTL-026* |
| *Crematogaster JTL-034* |
| *Crematogaster levior* |
| *Crematogaster limata* |
| *Crematogaster nigropilosa* |
| *Crematogaster rochai* |
| *Crematogaster sotobosque* |
| *Crematogaster stollii* |
| *Crematogaster tenuicula* |
| *Cylindromyrmex godmani* |
| *Cyphomyrmex cf. major sp. 1* |
| *Cyphomyrmex cf. minutus sp. 1* |
| *Cyphomyrmex cf. minutus sp. 2* |
| *Cyphomyrmex cf. rimosus* |
| *Cyphomyrmex costatus* |
| *Cyphomyrmex laevigatus* |
| *Cyphomyrmex sp. 2* |
| *Cyphomyrmex sp. 3* |
| *Cyphomyrmex vorticis* |
| *Discothyrea denticulata* |
| *Discothyrea horni* |
| *Discothyrea JSC-001* |
| *Discothyrea sexarticulata* |
| *Dolichoderus attelaboides* |
| *Dolichoderus bidens* |
| *Dolichoderus decollatus* |
| *Dolichoderus diversus* |
| *Dolichoderus imitator* |
| *Dolichoderus inpai* |
| *Dolichoderus lamellosus* |
| *Dolichoderus laminatus* |
| *Dolichoderus lobicornis* |
| *Dolichoderus lutosus* |
| *Dolichoderus quadridenticulatus* |
| *Dolichoderus rugosus* |
| *Dolichoderus shattucki* |
| *Dolichoderus validus* |
| *Dolichoderus varians* |
| *Dolopomyrmex n. sp.* |
| *Eciton hamatum* |
| *Eciton vagans* |
| *Ectatomma edentatum* |
| *Ectatomma lugens* |
| *Ectatomma tuberculatum* |
| *Gigantiops destructor* |
| *Gnamptogenys cf. sulcata* |
| *Gnamptogenys concinna* |
| *Gnamptogenys haenschi* |
| *Gnamptogenys horni* |
| *Gnamptogenys kempfi* |
| *Gnamptogenys KTRW-001* |
| *Gnamptogenys mediatrix* |
| *Gnamptogenys mina* |
| *Gnamptogenys minuta* |
| *Gnamptogenys moelleri* |
| *Gnamptogenys n. sp. A* |
| *Gnamptogenys n. sp. B* |
| *Gnamptogenys pleurodon* |
| *Gnamptogenys regularis* |
| *Gnamptogenys simulans* |
| *Gnamptogenys striatula* |
| *Gnamptogenys sulcata* |
| *Hylomyrma blandiens* |
| *Hylomyrma dolichops* |
| *Hylomyrma immanis* |
| *Hylomyrma praepotens* |
| *Hylomyrma sagax* |
| *Hypoponera c.f. creola* |
| *Hypoponera c.f. distinguenda* |
| *Hypoponera c.f. inexorata* |
| *Hypoponera c.f. parva* |
| *Hypoponera perplexa* |
| *Hypoponera STD 10* |
| *Hypoponera STD 11* |
| *Hypoponera STD 12* |
| *Hypoponera STD 13* |
| *Hypoponera STD 14* |
| *Hypoponera STD 15* |
| *Hypoponera STD 16* |
| *Hypoponera STD 17* |
| *Hypoponera STD 19* |
| *Hypoponera STD 20* |
| *Hypoponera STD 21* |
| *Hypoponera STD 22* |
| *Labidus coecus* |
| *Labidus praedator* |
| *Labidus punctaticeps* |
| *Lachnomyrmex scrobiculatus* |
| *Leptogenys gaigei* |
| *Leptogenys imperatrix* |
| *Leptogenys nigricans n. sp.* |
| *Leptogenys ritae* |
| *Megalomyrmex balzani* |
| *Megalomyrmex cuatiara* |
| *Megalomyrmex foreli* |
| *Megalomyrmex incisus* |
| *Megalomyrmex mondabora* |
| *Megalomyrmex n. sp. near drifti* |
| *Megalomyrmex silvestrii* |
| *Megalomyrmex timbira* |
| *Mycetarotes acutus* |
| *Mycetarotes unknown* |
| *Mycocepurus smithii* |
| *Myrmelachista KTRW-001* |
| *Myrmelachista KTRW-002* |
| *Myrmelachista KTRW-003* |
| *Myrmelachista KTRW-004* |
| *Myrmelachista KTRW-005* |
| *Myrmelachista KTRW-006* |
| *Myrmelachista KTRW-007* |
| *Myrmelachista KTRW-008* |
| *Myrmelachista KTRW-009* |
| *Myrmicocrypta cf. longinoda* |
| *Myrmicocrypta cf. rudiscapa* |
| *Myrmicocrypta longinoda* |
| *Neivamyrmex pseudops* |
| *Neivamyrmex punctaticeps* |
| *Nesomyrmex argentinus* |
| *Nesomyrmex brasiliensis* |
| *Nesomyrmex costatus* |
| *Nesomyrmex echinatinodis* |
| *Nesomyrmex pleuriticus* |
| *Nesomyrmex rutilans* |
| *Nesomyrmex spininodis* |
| *Nesomyrmex tristani* |
| *Nomamyrmex esenbecki* |
| *Nylanderia #11* |
| *Nylanderia #9* |
| *Nylanderia cf. fulva* |
| *Nylanderia cf. steinheili* |
| *Nylanderia KTRW001* |
| *Nylanderia KTRW002* |
| *Nylanderia KTRW003* |
| *Nylanderia KTRW004* |
| *Nylanderia KTRW005* |
| *Nylanderia KTRW006* |
| *Nylanderia KTRW008* |
| *Ochetomyrmex neopolitus* |
| *Ochetomyrmex semipolitus* |
| *Octostruma iheringi* |
| *Octostruma KTRW-002* |
| *Octostruma KTRW-003* |
| *Octostruma KTRW-004* |
| *Octostruma KTRW-005* |
| *Octostruma KTRW-006* |
| *Octostruma KTRW-007* |
| *Octostruma KTRW-008* |
| *Odontomachus biumbonatus* |
| *Odontomachus haematodus* |
| *Odontomachus hastatus* |
| *Odontomachus mayi* |
| *Odontomachus meinerti* |
| *Odontomachus panamensis* |
| *Odontomachus yucatecus* |
| *Oxyepoecus ephippiatus* |
| *Pachycondyla aenescens* |
| *Pachycondyla apicalis* |
| *Pachycondyla arhuaca* |
| *Pachycondyla carinulata* |
| *Pachycondyla cavinodis* |
| *Pachycondyla constricta* |
| *Pachycondyla crassinoda* |
| *Pachycondyla crenata* |
| *Pachycondyla foetida* |
| *Pachycondyla gilberti* |
| *Pachycondyla globosa* |
| *Pachycondyla harpax* |
| *Pachycondyla impressa* |
| *Pachycondyla inversa* |
| *Pachycondyla laevigata* |
| *Pachycondyla lunaris* |
| *Pachycondyla marginata* |
| *Pachycondyla oberthueri* |
| *Pachycondyla obscuricornis* |
| *Pachycondyla rostrata* |
| *Pachycondyla striatinodis* |
| *Pachycondyla unidentata* |
| *Pachycondyla verenae* |
| *Pachycondyla villosa* |
| *Paraponera clavata* |
| *Pheidole ademonia* |
| *Pheidole allarmata* |
| *Pheidole ALM-006* |
| *Pheidole ALM-013* |
| *Pheidole ALM-022* |
| *Pheidole ALM-023* |
| *Pheidole ALM-025* |
| *Pheidole ALM-026* |
| *Pheidole ALM-028* |
| *Pheidole ALM-031* |
| *Pheidole ALM-032* |
| *Pheidole ALM-033* |
| *Pheidole ALM-034* |
| *Pheidole amazonica* |
| *Pheidole araneoides* |
| *Pheidole astur* |
| *Pheidole biconstricta* |
| *Pheidole cephalica* |
| *Pheidole cramptoni* |
| *Pheidole deima* |
| *Pheidole exigua* |
| *Pheidole fimbriata* |
| *Pheidole floricola* |
| *Pheidole fracticeps* |
| *Pheidole gagates* |
| *Pheidole gilva* |
| *Pheidole horribilis* |
| *Pheidole laidlowi* |
| *Pheidole lemnisca* |
| *Pheidole metana* |
| *Pheidole midas* |
| *Pheidole nitella* |
| *Pheidole peruviana* |
| *Pheidole pholeops* |
| *Pheidole pubiventris* |
| *Pheidole sabella* |
| *Pheidole sagax* |
| *Pheidole sarpedon* |
| *Pheidole scalaris* |
| *Pheidole scolioceps* |
| *Pheidole tobini* |
| *Pheidole triplex* |
| *Pheidole tristicula* |
| *Pheidole xanthogaster* |
| *Platythyrea angusta* |
| *Prionopelta amabilis* |
| *Probolomyrmex petiolatus* |
| *Procryptocerus attenuatus* |
| *Procryptocerus coriarius* |
| *Procryptocerus hylaeus* |
| *Procryptocerus impressus* |
| *Procryptocerus n. sp. near eladio* |
| *Procryptocerus nalini* |
| *Procryptocerus paleatus* |
| *Procryptocerus pictipes* |
| *Pseudomyrmex atripes* |
| *Pseudomyrmex colei* |
| *Pseudomyrmex duckei* |
| *Pseudomyrmex eduardi* |
| *Pseudomyrmex elongatus* |
| *Pseudomyrmex ethicus* |
| *Pseudomyrmex faber* |
| *Pseudomyrmex filiformis* |
| *Pseudomyrmex gracilis* |
| *Pseudomyrmex laevifrons* |
| *Pseudomyrmex oculatus* |
| *Pseudomyrmex pupa* |
| *Pseudomyrmex rochai* |
| *Pseudomyrmex sericeus* |
| *Pseudomyrmex simplex* |
| *Pseudomyrmex sp. nr. cladoicus* |
| *Pseudomyrmex sp. nr. maculatus* |
| *Pseudomyrmex sp. nr. spiculus* |
| *Pseudomyrmex sp. PSW-161* |
| *Pseudomyrmex sp. PSW-37* |
| *Pseudomyrmex sp. PSW-52* |
| *Pseudomyrmex sp. PSW-58* |
| *Pseudomyrmex sp. PSW-59* |
| *Pseudomyrmex spiculus* |
| *Pseudomyrmex subater* |
| *Pseudomyrmex tenuis* |
| *Pseudomyrmex terminalis* |
| *Pseudomyrmex unicolor* |
| *Pseudomyrmex urbanus* |
| *Pseudomyrmex viduus* |
| *Pyramica beebei* |
| *Pyramica decipula* |
| *Pyramica denticulata* |
| *Pyramica depressiceps* |
| *Pyramica eggersi* |
| *Pyramica epinotalis* |
| *Pyramica glenognatha* |
| *Pyramica gundlachi* |
| *Pyramica metopia* |
| *Pyramica schulzi* |
| *Pyramica subedentata* |
| *Pyramica urrhobia* |
| *Pyramica villiersi* |
| *Pyramica zeteki* |
| *Rhopalothrix n. sp.1* |
| *Rhopalothrix n. sp.2* |
| *Rhopalothrix n.sp.3* |
| *Rogeria blanda* |
| *Rogeria ciliosa* |
| *Rogeria JSC-001* |
| *Rogeria JSC-002* |
| *Rogeria lirata* |
| *Rogeria micromma* |
| *Rogeria scobinata* |
| *Rogeria subarmata* |
| *Rogeria tonduzi* |
| *Rogeria unguispina* |
| *Sericomyrmex sp.1* |
| *Sericomyrmex sp.2* |
| *Solenopsis SC-02* |
| *Solenopsis SC-03* |
| *Solenopsis SC-05* |
| *Solenopsis SC-06* |
| *Solenopsis SC-08* |
| *Solenopsis SC-09* |
| *Solenopsis SC-10* |
| *Solenopsis SC-11* |
| *Solenopsis SC-12* |
| *Solenopsis SC-13* |
| *Solenopsis SC-14* |
| *Solenopsis SC-15* |
| *Solenopsis SC-16* |
| *Solenopsis SC-17* |
| *Solenopsis virulens* |
| *Stegomyrmex connectens* |
| *Stegomyrmex manni* |
| *Strumigenys cosmostela* |
| *Strumigenys dolichognatha* |
| *Strumigenys elongata* |
| *Strumigenys incuba* |
| *Strumigenys perparva* |
| *Strumigenys precava* |
| *Strumigenys smithii* |
| *Strumigenys tococae* |
| *Strumigenys trinidadensis* |
| *Strumigenys trudifera* |
| *Strumigenys vilhenai* |
| *Tapinoma KTRW-001* |
| *Tapinoma KTRW-002* |
| *Tapinoma KTRW-003* |
| *Tapinoma KTRW-004* |
| *Trachymyrmex cf. bugnioni* |
| *Trachymyrmex cf. opulentus* |
| *Trachymyrmex diversus* |
| *Trachymyrmex farinosus* |
| *Trachymyrmex ruthae* |
| *Tranopelta gilva* |
| *Tranopelta n. sp.* |
| *Tranopelta subterranea* |
| *Typhlomyrmex pusillus* |
| *Typhlomyrmex rogenhoferi* |
| *Wasmannia auropunctata* |
| *Wasmannia cf. lutzi* |
| *Wasmannia iheringi* |
| *Wasmannia rochai* |
| *Wasmannia scrobifera* |
