# Supplementary material for: Species Diversity and Distribution Patterns of the Ants of Amazonian Ecuador
Source: PLoS One. 2010 Oct 1;5(10):e13146. doi: 10.1371/journal.pone.0013146 (PMC2948521; doi:10.1371/journal.pone.0013146)
Supplement: Table S2 — Summary of results of sampling methods. (0.03 MB DOC) [file pone.0013146.s002.doc]

**Table S2.** Summary of results of sampling methods. **†** Ants are social, and entire colonies may occur in single samples. To reduce the effect of this extreme spatial aggregation, only incidence data are used in this report. ‡ Number of species found in only one sample for a particular method, but not entire study [following terminology of 92]. **§** Number of species found in only one sample in the entire study.

| **Collection Method** | **No. Samples** | **No. Species Occurrences†** | **No. Species** | **No. Local Uniques‡** | **No. Uniques§** | **Most Abundant Genera** | **Most Diverse Genera** |
| --- | --- | --- | --- | --- | --- | --- | --- |
| Canopy | 100 | 3352 | 282 | 92 | 55 | *Crematogaster Camponotus Solenopsis Pachycondyla* | *Camponotus Pseudomyrmex Crematogaster Pachycondyla* |
| Hand collected | 60 | 965 | 150 | 63 | 9 | *Pheidole Crematogaster Pachycondyla Wasmannia* | *Pheidole Pachycondyla Crematogaster Camponotus Hypoponera* |
| Bait | 60 | 423 | 83 | 34 | 1 | *Pheidole Megalomyrmex Trachymyrmex Camponotus* | *Pheidole  Solenopsis Trachymyrmex Nylanderia Camponotus* |
| Pitfall | 60 | 618 | 96 | 30 | 4 | *Pheidole Pachycondyla Gnamptogenys Camponotus Crematogaster* | *Pheidole  Solenopsis Pachycondyla Crematogaster Gnamptogenys* |
| Mini-Winkler | 60 | 1943 | 185 | 69 | 24 | *Pheidole  Pyramica Hypoponera Gnamptogenys* | *Pheidole Hypoponera Pyramica  Solenopsis* |
| Subterranean probe | 50 | 438 | 42 | 9 | 0 | *Labidus  Solenopsis  Pheidole  Tranopelta* | *Pheidole  Solenopsis* |
| **Total** | **390** | **7740** | **475** |  | **93** | *Pheidole Camponotus Crematogaster Solenopsis Pachycondyla* | *Camponotus Pheidole Pseudomyrmex Pachycondyla* |
