# Supplementary material for: Species Diversity and Distribution Patterns of the Ants of Amazonian Ecuador
Source: PLoS One. 2010 Oct 1;5(10):e13146. doi: 10.1371/journal.pone.0013146 (PMC2948521; doi:10.1371/journal.pone.0013146)
Supplement: Table S3 — Abundance (number of occurrences in each sample) by sampling method of each species. (0.67 MB DOC) [file pone.0013146.s003.doc]

**Table S3.** Abundance (number of occurrences in each sample) by sampling method of each species.

|  | **Canopy** | **Ground** | **Subterranean** | **Total** |
| --- | --- | --- | --- | --- |
| *Acanthoponera minor* | 3 | 0 | 0 | 3 |
| *Acanthoponera peruviana* | 9 | 0 | 0 | 9 |
| *Acanthostichus quadratus* | 0 | 0 | 17 | 17 |
| *Acromyrmex coronatus* | 6 | 14 | 0 | 20 |
| *Acropyga decedens* | 0 | 9 | 0 | 9 |
| *Acropyga donisthorpei* | 0 | 3 | 0 | 3 |
| *Acropyga fuhrmanni* | 0 | 6 | 0 | 6 |
| *Acropyga guianensis* | 0 | 6 | 0 | 6 |
| *Amblyopone cf. cleae* | 0 | 1 | 0 | 1 |
| *Anochetus bispinosus* | 6 | 0 | 0 | 6 |
| *Anochetus diegensis* | 0 | 19 | 0 | 19 |
| *Anochetus mayri* | 0 | 17 | 0 | 17 |
| *Apterostigma auriculatum* | 0 | 5 | 0 | 5 |
| *Apterostigma sp.1* | 1 | 0 | 0 | 1 |
| *Apterostigma sp.2* | 8 | 3 | 0 | 11 |
| *Apterostigma sp.3* | 0 | 4 | 0 | 4 |
| *Apterostigma sp.4* | 0 | 7 | 0 | 7 |
| *Apterostigma sp.5* | 0 | 1 | 0 | 1 |
| *Apterostigma sp.6* | 0 | 2 | 0 | 2 |
| *Apterostigma sp.7* | 0 | 1 | 0 | 1 |
| *Azteca SJ-A* | 33 | 1 | 0 | 34 |
| *Azteca SJ-AA* | 17 | 1 | 0 | 18 |
| *Azteca SJ-B* | 11 | 1 | 0 | 12 |
| *Azteca SJ-BB* | 4 | 0 | 0 | 4 |
| *Azteca SJ-C* | 9 | 0 | 0 | 9 |
| *Azteca SJ-CC* | 3 | 0 | 0 | 3 |
| *Azteca SJ-D* | 1 | 0 | 0 | 1 |
| *Azteca SJ-DD* | 1 | 0 | 0 | 1 |
| *Azteca SJ-E* | 15 | 0 | 0 | 15 |
| *Azteca SJ-EE* | 1 | 0 | 0 | 1 |
| *Azteca SJ-F* | 18 | 0 | 0 | 18 |
| *Azteca SJ-FF* | 3 | 0 | 0 | 3 |
| *Azteca SJ-G* | 39 | 0 | 0 | 39 |
| *Azteca SJ-GG* | 10 | 0 | 0 | 10 |
| *Azteca SJ-H* | 2 | 0 | 0 | 2 |
| *Azteca SJ-HH* | 1 | 0 | 0 | 1 |
| *Azteca SJ-I* | 2 | 1 | 0 | 3 |
| *Azteca SJ-II* | 1 | 0 | 0 | 1 |
| *Azteca SJ-J* | 1 | 0 | 0 | 1 |
| *Azteca SJ-JJ* | 5 | 0 | 0 | 5 |
| *Azteca SJ-KK* | 1 | 0 | 0 | 1 |
| *Azteca SJ-LL* | 1 | 0 | 0 | 1 |
| *Azteca SJ-M* | 0 | 1 | 0 | 1 |
| *Azteca SJ-MM* | 5 | 0 | 0 | 5 |
| *Azteca SJ-N* | 3 | 0 | 0 | 3 |
| *Azteca SJ-NN* | 1 | 0 | 0 | 1 |
| *Azteca SJ-O* | 2 | 0 | 0 | 2 |
| *Azteca SJ-OO* | 1 | 0 | 0 | 1 |
| *Azteca SJ-P* | 0 | 2 | 0 | 2 |
| *Azteca SJ-PP* | 3 | 0 | 0 | 3 |
| *Azteca SJ-Q* | 1 | 0 | 0 | 1 |
| *Azteca SJ-R* | 1 | 0 | 0 | 1 |
| *Basiceros conjugans* | 0 | 12 | 0 | 12 |
| *Basiceros manni* | 0 | 1 | 0 | 1 |
| *Basiceros militaris* | 0 | 16 | 0 | 16 |
| *Brachymyrmex cavernicola* | 1 | 38 | 10 | 49 |
| *Brachymyrmex KTRW-001* | 0 | 11 | 13 | 24 |
| *Brachymyrmex KTRW-002* | 2 | 0 | 0 | 2 |
| *Brachymyrmex KTRW-003* | 8 | 0 | 0 | 8 |
| *Brachymyrmex KTRW-004* | 1 | 0 | 0 | 1 |
| *Brachymyrmex KTRW-005* | 0 | 1 | 0 | 1 |
| *Brachymyrmex KTRW-006* | 1 | 0 | 0 | 1 |
| *Brachymyrmex KTRW-007* | 0 | 8 | 0 | 8 |
| *Brachymyrmex KTRW-008* | 2 | 0 | 0 | 2 |
| *Brachymyrmex KTRW-009* | 4 | 0 | 0 | 4 |
| *Brachymyrmex KTRW-010* | 1 | 0 | 0 | 1 |
| *Brachymyrmex KTRW-011* | 1 | 0 | 0 | 1 |
| *Brachymyrmex KTRW-012* | 7 | 0 | 0 | 7 |
| *Brachymyrmex KTRW-013* | 1 | 0 | 0 | 1 |
| *Brachymyrmex KTRW-014* | 1 | 7 | 0 | 8 |
| *Brachymyrmex KTRW-015* | 0 | 7 | 0 | 7 |
| *Brachymyrmex KTRW-016* | 2 | 7 | 0 | 9 |
| *Brachymyrmex KTRW-017* | 1 | 0 | 0 | 1 |
| *Brachymyrmex KTRW-018* | 1 | 0 | 0 | 1 |
| *Brachymyrmex KTRW-019* | 53 | 0 | 0 | 53 |
| *Camponotus abscisus* | 5 | 0 | 0 | 5 |
| *Camponotus arboreus* | 2 | 0 | 0 | 2 |
| *Camponotus atriceps* | 83 | 6 | 0 | 89 |
| *Camponotus bidens* | 55 | 0 | 0 | 55 |
| *Camponotus bispinosus* | 0 | 2 | 0 | 2 |
| *Camponotus bradleyi* | 1 | 0 | 0 | 1 |
| *Camponotus branneri* | 1 | 0 | 0 | 1 |
| *Camponotus brevis* | 2 | 0 | 0 | 2 |
| *Camponotus cacicus* | 4 | 4 | 0 | 8 |
| *Camponotus callistus* | 2 | 0 | 0 | 2 |
| *Camponotus claviscapus* | 38 | 2 | 0 | 40 |
| *Camponotus constructor* | 10 | 0 | 0 | 10 |
| *Camponotus emeryodicatus* | 1 | 0 | 0 | 1 |
| *Camponotus eurynotus* | 7 | 0 | 0 | 7 |
| *Camponotus excisus* | 79 | 0 | 0 | 79 |
| *Camponotus femoratus* | 55 | 82 | 24 | 161 |
| *Camponotus formiciformis* | 19 | 0 | 0 | 19 |
| *Camponotus helleri* | 3 | 0 | 0 | 3 |
| *Camponotus hippocrepis* | 7 | 0 | 0 | 7 |
| *Camponotus integellus* | 10 | 4 | 0 | 14 |
| *Camponotus latangulus* | 14 | 0 | 0 | 14 |
| *Camponotus linnaei* | 1 | 0 | 0 | 1 |
| *Camponotus macrochaeta* | 1 | 0 | 0 | 1 |
| *Camponotus mocsaryi* | 1 | 0 | 0 | 1 |
| *Camponotus mus* | 8 | 0 | 0 | 8 |
| *Camponotus nidulans* | 4 | 1 | 0 | 5 |
| *Camponotus nitidior* | 7 | 0 | 0 | 7 |
| *Camponotus novogranadensis* | 3 | 0 | 0 | 3 |
| *Camponotus orthocephalus* | 2 | 0 | 0 | 2 |
| *Camponotus planatus* | 0 | 1 | 0 | 1 |
| *Camponotus rapax* | 3 | 2 | 4 | 9 |
| *Camponotus senex* | 18 | 1 | 0 | 19 |
| *Camponotus sexguttatus* | 30 | 0 | 0 | 30 |
| *Camponotus WM-001* | 3 | 0 | 0 | 3 |
| *Camponotus WM-002* | 3 | 0 | 0 | 3 |
| *Camponotus WM-003* | 3 | 0 | 0 | 3 |
| *Camponotus WM-004* | 1 | 0 | 0 | 1 |
| *Camponotus WM-005* | 4 | 0 | 0 | 4 |
| *Camponotus WM-006* | 7 | 0 | 0 | 7 |
| *Camponotus WM-007* | 3 | 0 | 0 | 3 |
| *Camponotus WM-008* | 14 | 1 | 0 | 15 |
| *Camponotus WM-009* | 1 | 0 | 0 | 1 |
| *Camponotus WM-010* | 0 | 6 | 0 | 6 |
| *Camponotus wytsmani* | 2 | 0 | 0 | 2 |
| *Carebara angulata* | 0 | 1 | 0 | 1 |
| *Carebara panamensis* | 0 | 3 | 0 | 3 |
| *Carebara paya* | 0 | 1 | 17 | 18 |
| *Carebara urichi* | 0 | 5 | 8 | 13 |
| *Carebarella KTRW-001* | 0 | 16 | 0 | 16 |
| *Centromyrmex alfaroi* | 0 | 0 | 2 | 2 |
| *Cephalotes atratus* | 36 | 0 | 0 | 36 |
| *Cephalotes cordatus* | 1 | 0 | 0 | 1 |
| *Cephalotes laminatus* | 6 | 0 | 0 | 6 |
| *Cephalotes maculatus* | 12 | 0 | 0 | 12 |
| *Cephalotes manni* | 4 | 0 | 0 | 4 |
| *Cephalotes marginatus* | 1 | 0 | 0 | 1 |
| *Cephalotes minutus* | 16 | 3 | 0 | 19 |
| *Cephalotes n. sp. near maculatus* | 1 | 0 | 0 | 1 |
| *Cephalotes n. sp. near palta* | 1 | 0 | 0 | 1 |
| *Cephalotes opacus* | 40 | 0 | 0 | 40 |
| *Cephalotes pallidus* | 7 | 0 | 0 | 7 |
| *Cephalotes pavonii* | 4 | 0 | 0 | 4 |
| *Cephalotes peruviensis* | 13 | 0 | 0 | 13 |
| *Cephalotes ramiphilus* | 1 | 0 | 0 | 1 |
| *Cephalotes spinosus* | 6 | 0 | 0 | 6 |
| *Cephalotes umbraculatus* | 3 | 0 | 0 | 3 |
| *Crematogaster acuta* | 0 | 2 | 0 | 2 |
| *Crematogaster brasiliensis* | 113 | 2 | 0 | 115 |
| *Crematogaster carinata* | 237 | 34 | 0 | 271 |
| *Crematogaster crucis* | 3 | 0 | 0 | 3 |
| *Crematogaster curvispinosa* | 15 | 0 | 0 | 15 |
| *Crematogaster egregior* | 13 | 0 | 0 | 13 |
| *Crematogaster erecta* | 42 | 6 | 0 | 48 |
| *Crematogaster flavomicrops* | 2 | 9 | 0 | 11 |
| *Crematogaster foliocrypta* | 2 | 0 | 0 | 2 |
| *Crematogaster JTL-022* | 20 | 0 | 0 | 20 |
| *Crematogaster JTL-026* | 8 | 0 | 0 | 8 |
| *Crematogaster JTL-034* | 9 | 0 | 0 | 9 |
| *Crematogaster levior* | 59 | 42 | 7 | 108 |
| *Crematogaster limata* | 50 | 21 | 0 | 71 |
| *Crematogaster nigropilosa* | 0 | 3 | 0 | 3 |
| *Crematogaster rochai* | 1 | 0 | 0 | 1 |
| *Crematogaster sotobosque* | 1 | 28 | 0 | 29 |
| *Crematogaster stollii* | 2 | 6 | 0 | 8 |
| *Crematogaster tenuicula* | 16 | 1 | 0 | 17 |
| *Cylindromyrmex godmani* | 1 | 0 | 0 | 1 |
| *Cyphomyrmex cf. major sp. 1* | 29 | 0 | 0 | 29 |
| *Cyphomyrmex cf. minutus sp. 1* | 0 | 43 | 0 | 43 |
| *Cyphomyrmex cf. minutus sp. 2* | 0 | 10 | 0 | 10 |
| *Cyphomyrmex cf. rimosus* | 1 | 26 | 0 | 27 |
| *Cyphomyrmex costatus* | 0 | 1 | 0 | 1 |
| *Cyphomyrmex laevigatus* | 0 | 11 | 0 | 11 |
| *Cyphomyrmex sp. 2* | 0 | 2 | 0 | 2 |
| *Cyphomyrmex sp. 3* | 0 | 2 | 0 | 2 |
| *Cyphomyrmex vorticis* | 0 | 9 | 0 | 9 |
| *Discothyrea denticulata* | 0 | 7 | 0 | 7 |
| *Discothyrea horni* | 1 | 1 | 0 | 2 |
| *Discothyrea JSC-001* | 0 | 3 | 0 | 3 |
| *Discothyrea sexarticulata* | 0 | 8 | 0 | 8 |
| *Dolichoderus attelaboides* | 13 | 0 | 0 | 13 |
| *Dolichoderus bidens* | 20 | 0 | 0 | 20 |
| *Dolichoderus decollatus* | 12 | 0 | 0 | 12 |
| *Dolichoderus diversus* | 21 | 0 | 0 | 21 |
| *Dolichoderus imitator* | 39 | 6 | 0 | 45 |
| *Dolichoderus inpai* | 5 | 4 | 0 | 9 |
| *Dolichoderus lamellosus* | 3 | 0 | 0 | 3 |
| *Dolichoderus laminatus* | 1 | 0 | 0 | 1 |
| *Dolichoderus lobicornis* | 1 | 0 | 0 | 1 |
| *Dolichoderus lutosus* | 1 | 0 | 0 | 1 |
| *Dolichoderus quadridenticulatus* | 5 | 0 | 0 | 5 |
| *Dolichoderus rugosus* | 8 | 6 | 0 | 14 |
| *Dolichoderus shattucki* | 0 | 1 | 0 | 1 |
| *Dolichoderus validus* | 3 | 0 | 0 | 3 |
| *Dolichoderus varians* | 5 | 0 | 0 | 5 |
| *Dolopomyrmex n. sp.* | 0 | 0 | 22 | 22 |
| *Eciton hamatum* | 0 | 6 | 0 | 6 |
| *Eciton vagans* | 0 | 2 | 0 | 2 |
| *Ectatomma edentatum* | 0 | 9 | 0 | 9 |
| *Ectatomma lugens* | 0 | 26 | 0 | 26 |
| *Ectatomma tuberculatum* | 40 | 1 | 0 | 41 |
| *Gigantiops destructor* | 11 | 5 | 0 | 16 |
| *Gnamptogenys cf. sulcata* | 2 | 0 | 0 | 2 |
| *Gnamptogenys concinna* | 2 | 0 | 0 | 2 |
| *Gnamptogenys haenschi* | 0 | 1 | 4 | 5 |
| *Gnamptogenys horni* | 0 | 41 | 0 | 41 |
| *Gnamptogenys kempfi* | 0 | 1 | 0 | 1 |
| *Gnamptogenys KTRW-001* | 0 | 50 | 0 | 50 |
| *Gnamptogenys mediatrix* | 0 | 2 | 0 | 2 |
| *Gnamptogenys mina* | 0 | 5 | 0 | 5 |
| *Gnamptogenys minuta* | 0 | 2 | 0 | 2 |
| *Gnamptogenys moelleri* | 0 | 4 | 0 | 4 |
| *Gnamptogenys n. sp. A* | 1 | 0 | 0 | 1 |
| *Gnamptogenys n. sp. B* | 2 | 0 | 0 | 2 |
| *Gnamptogenys pleurodon* | 27 | 27 | 0 | 54 |
| *Gnamptogenys regularis* | 1 | 0 | 0 | 1 |
| *Gnamptogenys simulans* | 0 | 3 | 0 | 3 |
| *Gnamptogenys striatula* | 0 | 52 | 0 | 52 |
| *Gnamptogenys sulcata* | 0 | 2 | 0 | 2 |
| *Hylomyrma blandiens* | 0 | 4 | 0 | 4 |
| *Hylomyrma dolichops* | 0 | 16 | 0 | 16 |
| *Hylomyrma immanis* | 0 | 28 | 0 | 28 |
| *Hylomyrma praepotens* | 0 | 5 | 0 | 5 |
| *Hylomyrma sagax* | 0 | 8 | 0 | 8 |
| *Hypoponera c.f. creola* | 0 | 51 | 0 | 51 |
| *Hypoponera c.f. distinguenda* | 0 | 29 | 0 | 29 |
| *Hypoponera c.f. inexorata* | 0 | 2 | 0 | 2 |
| *Hypoponera c.f. parva* | 0 | 19 | 0 | 19 |
| *Hypoponera perplexa* | 0 | 6 | 0 | 6 |
| *Hypoponera STD 10* | 0 | 10 | 0 | 10 |
| *Hypoponera STD 11* | 0 | 9 | 0 | 9 |
| *Hypoponera STD 12* | 0 | 1 | 0 | 1 |
| *Hypoponera STD 13* | 0 | 6 | 0 | 6 |
| *Hypoponera STD 14* | 0 | 1 | 0 | 1 |
| *Hypoponera STD 15* | 0 | 2 | 0 | 2 |
| *Hypoponera STD 16* | 0 | 1 | 0 | 1 |
| *Hypoponera STD 17* | 0 | 5 | 0 | 5 |
| *Hypoponera STD 19* | 3 | 0 | 0 | 3 |
| *Hypoponera STD 20* | 0 | 2 | 0 | 2 |
| *Hypoponera STD 21* | 0 | 9 | 0 | 9 |
| *Hypoponera STD 22* | 0 | 9 | 0 | 9 |
| *Labidus coecus* | 0 | 4 | 66 | 70 |
| *Labidus praedator* | 0 | 2 | 0 | 2 |
| *Labidus punctaticeps* | 0 | 10 | 0 | 10 |
| *Lachnomyrmex scrobiculatus* | 0 | 1 | 0 | 1 |
| *Leptogenys gaigei* | 0 | 1 | 0 | 1 |
| *Leptogenys imperatrix* | 0 | 2 | 0 | 2 |
| *Leptogenys nigricans n. sp.* | 0 | 5 | 0 | 5 |
| *Leptogenys ritae* | 0 | 1 | 0 | 1 |
| *Megalomyrmex balzani* | 2 | 7 | 0 | 9 |
| *Megalomyrmex cuatiara* | 0 | 2 | 0 | 2 |
| *Megalomyrmex foreli* | 16 | 100 | 4 | 120 |
| *Megalomyrmex incisus* | 0 | 10 | 0 | 10 |
| *Megalomyrmex mondabora* | 0 | 1 | 0 | 1 |
| *Megalomyrmex n. sp. near drifti* | 0 | 3 | 0 | 3 |
| *Megalomyrmex silvestrii* | 0 | 2 | 0 | 2 |
| *Megalomyrmex timbira* | 1 | 2 | 0 | 3 |
| *Mycetarotes acutus* | 0 | 1 | 0 | 1 |
| *Mycetarotes unknown* | 0 | 2 | 0 | 2 |
| *Mycocepurus smithii* | 0 | 18 | 0 | 18 |
| *Myrmelachista KTRW-001* | 6 | 5 | 0 | 11 |
| *Myrmelachista KTRW-002* | 5 | 0 | 0 | 5 |
| *Myrmelachista KTRW-003* | 6 | 1 | 0 | 7 |
| *Myrmelachista KTRW-004* | 2 | 0 | 0 | 2 |
| *Myrmelachista KTRW-005* | 1 | 0 | 0 | 1 |
| *Myrmelachista KTRW-006* | 6 | 0 | 0 | 6 |
| *Myrmelachista KTRW-007* | 14 | 0 | 0 | 14 |
| *Myrmelachista KTRW-008* | 1 | 0 | 0 | 1 |
| *Myrmelachista KTRW-009* | 1 | 0 | 0 | 1 |
| *Myrmicocrypta cf. longinoda* | 1 | 0 | 0 | 1 |
| *Myrmicocrypta cf. rudiscapa* | 1 | 1 | 0 | 2 |
| *Myrmicocrypta longinoda* | 0 | 21 | 0 | 21 |
| *Neivamyrmex pseudops* | 0 | 2 | 0 | 2 |
| *Neivamyrmex punctaticeps* | 0 | 0 | 22 | 22 |
| *Nesomyrmex argentinus* | 4 | 0 | 0 | 4 |
| *Nesomyrmex brasiliensis* | 2 | 0 | 0 | 2 |
| *Nesomyrmex costatus* | 1 | 0 | 0 | 1 |
| *Nesomyrmex echinatinodis* | 7 | 0 | 0 | 7 |
| *Nesomyrmex pleuriticus* | 21 | 0 | 0 | 21 |
| *Nesomyrmex rutilans* | 3 | 0 | 0 | 3 |
| *Nesomyrmex spininodis* | 24 | 0 | 0 | 24 |
| *Nesomyrmex tristani* | 2 | 0 | 0 | 2 |
| *Nomamyrmex esenbecki* | 0 | 5 | 0 | 5 |
| *Nylanderia #11* | 0 | 1 | 0 | 1 |
| *Nylanderia #9* | 0 | 2 | 0 | 2 |
| *Nylanderia cf. fulva* | 0 | 10 | 1 | 11 |
| *Nylanderia cf. steinheili* | 35 | 76 | 5 | 116 |
| *Nylanderia KTRW001* | 37 | 5 | 0 | 42 |
| *Nylanderia KTRW002* | 15 | 0 | 0 | 15 |
| *Nylanderia KTRW003* | 1 | 9 | 0 | 10 |
| *Nylanderia KTRW004* | 2 | 22 | 5 | 29 |
| *Nylanderia KTRW005* | 6 | 3 | 0 | 9 |
| *Nylanderia KTRW006* | 14 | 0 | 0 | 14 |
| *Nylanderia KTRW008* | 0 | 2 | 0 | 2 |
| *Ochetomyrmex neopolitus* | 1 | 13 | 0 | 14 |
| *Ochetomyrmex semipolitus* | 0 | 10 | 2 | 12 |
| *Octostruma iheringi* | 0 | 3 | 0 | 3 |
| *Octostruma KTRW-002* | 0 | 1 | 0 | 1 |
| *Octostruma KTRW-003* | 0 | 1 | 0 | 1 |
| *Octostruma KTRW-004* | 0 | 2 | 0 | 2 |
| *Octostruma KTRW-005* | 0 | 1 | 0 | 1 |
| *Octostruma KTRW-006* | 0 | 24 | 0 | 24 |
| *Octostruma KTRW-007* | 0 | 10 | 0 | 10 |
| *Octostruma KTRW-008* | 0 | 2 | 0 | 2 |
| *Odontomachus biumbonatus* | 0 | 2 | 2 | 4 |
| *Odontomachus haematodus* | 6 | 11 | 0 | 17 |
| *Odontomachus hastatus* | 2 | 0 | 0 | 2 |
| *Odontomachus mayi* | 8 | 0 | 0 | 8 |
| *Odontomachus meinerti* | 1 | 55 | 0 | 56 |
| *Odontomachus panamensis* | 0 | 3 | 0 | 3 |
| *Odontomachus yucatecus* | 0 | 3 | 0 | 3 |
| *Oxyepoecus ephippiatus* | 0 | 2 | 0 | 2 |
| *Pachycondyla aenescens* | 2 | 0 | 0 | 2 |
| *Pachycondyla apicalis* | 1 | 2 | 0 | 3 |
| *Pachycondyla arhuaca* | 1 | 4 | 0 | 5 |
| *Pachycondyla carinulata* | 19 | 0 | 0 | 19 |
| *Pachycondyla cavinodis* | 25 | 0 | 0 | 25 |
| *Pachycondyla constricta* | 1 | 53 | 0 | 54 |
| *Pachycondyla crassinoda* | 0 | 68 | 2 | 70 |
| *Pachycondyla crenata* | 75 | 0 | 0 | 75 |
| *Pachycondyla foetida* | 4 | 0 | 0 | 4 |
| *Pachycondyla gilberti* | 0 | 1 | 0 | 1 |
| *Pachycondyla globosa* | 28 | 0 | 0 | 28 |
| *Pachycondyla harpax* | 1 | 62 | 0 | 63 |
| *Pachycondyla impressa* | 1 | 0 | 3 | 4 |
| *Pachycondyla inversa* | 20 | 1 | 0 | 21 |
| *Pachycondyla laevigata* | 0 | 2 | 0 | 2 |
| *Pachycondyla lunaris* | 0 | 1 | 0 | 1 |
| *Pachycondyla marginata* | 0 | 1 | 0 | 1 |
| *Pachycondyla oberthueri* | 1 | 0 | 0 | 1 |
| *Pachycondyla obscuricornis* | 0 | 2 | 0 | 2 |
| *Pachycondyla rostrata* | 3 | 0 | 0 | 3 |
| *Pachycondyla striatinodis* | 12 | 0 | 0 | 12 |
| *Pachycondyla unidentata* | 13 | 0 | 0 | 13 |
| *Pachycondyla verenae* | 0 | 6 | 0 | 6 |
| *Pachycondyla villosa* | 15 | 0 | 0 | 15 |
| *Paraponera clavata* | 43 | 1 | 0 | 44 |
| *Pheidole ademonia* | 0 | 2 | 0 | 2 |
| *Pheidole allarmata* | 0 | 24 | 0 | 24 |
| *Pheidole ALM-006* | 0 | 33 | 0 | 33 |
| *Pheidole ALM-013* | 0 | 6 | 0 | 6 |
| *Pheidole ALM-022* | 1 | 0 | 0 | 1 |
| *Pheidole ALM-023* | 1 | 0 | 0 | 1 |
| *Pheidole ALM-025* | 1 | 14 | 0 | 15 |
| *Pheidole ALM-026* | 0 | 1 | 0 | 1 |
| *Pheidole ALM-028* | 0 | 10 | 0 | 10 |
| *Pheidole ALM-031* | 0 | 5 | 0 | 5 |
| *Pheidole ALM-032* | 0 | 1 | 0 | 1 |
| *Pheidole ALM-033* | 10 | 0 | 0 | 10 |
| *Pheidole ALM-034* | 2 | 0 | 0 | 2 |
| *Pheidole amazonica* | 0 | 58 | 12 | 70 |
| *Pheidole araneoides* | 0 | 4 | 0 | 4 |
| *Pheidole astur* | 0 | 104 | 0 | 104 |
| *Pheidole biconstricta* | 20 | 52 | 8 | 80 |
| *Pheidole cephalica* | 0 | 7 | 0 | 7 |
| *Pheidole cramptoni* | 1 | 1 | 0 | 2 |
| *Pheidole deima* | 0 | 25 | 0 | 25 |
| *Pheidole exigua* | 0 | 1 | 0 | 1 |
| *Pheidole fimbriata* | 0 | 17 | 24 | 41 |
| *Pheidole floricola* | 3 | 0 | 0 | 3 |
| *Pheidole fracticeps* | 0 | 8 | 0 | 8 |
| *Pheidole gagates* | 0 | 1 | 0 | 1 |
| *Pheidole gilva* | 20 | 0 | 0 | 20 |
| *Pheidole horribilis* | 0 | 28 | 0 | 28 |
| *Pheidole laidlowi* | 0 | 1 | 0 | 1 |
| *Pheidole lemnisca* | 0 | 6 | 0 | 6 |
| *Pheidole metana* | 0 | 13 | 0 | 13 |
| *Pheidole midas* | 0 | 55 | 0 | 55 |
| *Pheidole nitella* | 0 | 87 | 6 | 93 |
| *Pheidole peruviana* | 0 | 17 | 2 | 19 |
| *Pheidole pholeops* | 1 | 16 | 0 | 17 |
| *Pheidole pubiventris* | 8 | 0 | 0 | 8 |
| *Pheidole sabella* | 0 | 6 | 0 | 6 |
| *Pheidole sagax* | 0 | 10 | 5 | 15 |
| *Pheidole sarpedon* | 0 | 2 | 0 | 2 |
| *Pheidole scalaris* | 0 | 4 | 0 | 4 |
| *Pheidole scolioceps* | 0 | 9 | 0 | 9 |
| *Pheidole tobini* | 0 | 7 | 0 | 7 |
| *Pheidole triplex* | 0 | 30 | 0 | 30 |
| *Pheidole tristicula* | 0 | 5 | 0 | 5 |
| *Pheidole xanthogaster* | 0 | 23 | 0 | 23 |
| *Platythyrea angusta* | 24 | 0 | 0 | 24 |
| *Prionopelta amabilis* | 0 | 3 | 0 | 3 |
| *Probolomyrmex petiolatus* | 0 | 3 | 0 | 3 |
| *Procryptocerus attenuatus* | 16 | 0 | 0 | 16 |
| *Procryptocerus coriarius* | 1 | 0 | 0 | 1 |
| *Procryptocerus hylaeus* | 6 | 0 | 0 | 6 |
| *Procryptocerus impressus* | 2 | 0 | 0 | 2 |
| *Procryptocerus n. sp. near eladio* | 1 | 0 | 0 | 1 |
| *Procryptocerus nalini* | 4 | 0 | 0 | 4 |
| *Procryptocerus paleatus* | 1 | 0 | 0 | 1 |
| *Procryptocerus pictipes* | 16 | 0 | 0 | 16 |
| *Pseudomyrmex atripes* | 3 | 0 | 0 | 3 |
| *Pseudomyrmex colei* | 1 | 0 | 0 | 1 |
| *Pseudomyrmex duckei* | 1 | 0 | 0 | 1 |
| *Pseudomyrmex eduardi* | 1 | 0 | 0 | 1 |
| *Pseudomyrmex elongatus* | 4 | 0 | 0 | 4 |
| *Pseudomyrmex ethicus* | 5 | 0 | 0 | 5 |
| *Pseudomyrmex faber* | 14 | 0 | 0 | 14 |
| *Pseudomyrmex filiformis* | 9 | 0 | 0 | 9 |
| *Pseudomyrmex gracilis* | 19 | 0 | 0 | 19 |
| *Pseudomyrmex laevifrons* | 3 | 0 | 0 | 3 |
| *Pseudomyrmex oculatus* | 24 | 0 | 0 | 24 |
| *Pseudomyrmex pupa* | 3 | 0 | 0 | 3 |
| *Pseudomyrmex rochai* | 15 | 0 | 0 | 15 |
| *Pseudomyrmex sericeus* | 1 | 0 | 0 | 1 |
| *Pseudomyrmex simplex* | 11 | 0 | 0 | 11 |
| *Pseudomyrmex sp. nr. cladoicus* | 1 | 0 | 0 | 1 |
| *Pseudomyrmex sp. nr. maculatus* | 1 | 0 | 0 | 1 |
| *Pseudomyrmex sp. nr. spiculus* | 1 | 0 | 0 | 1 |
| *Pseudomyrmex sp. PSW-161* | 1 | 0 | 0 | 1 |
| *Pseudomyrmex sp. PSW-37* | 6 | 0 | 0 | 6 |
| *Pseudomyrmex sp. PSW-52* | 1 | 0 | 0 | 1 |
| *Pseudomyrmex sp. PSW-58* | 2 | 0 | 0 | 2 |
| *Pseudomyrmex sp. PSW-59* | 1 | 0 | 0 | 1 |
| *Pseudomyrmex spiculus* | 1 | 0 | 0 | 1 |
| *Pseudomyrmex subater* | 1 | 0 | 0 | 1 |
| *Pseudomyrmex tenuis* | 6 | 1 | 0 | 7 |
| *Pseudomyrmex terminalis* | 12 | 0 | 0 | 12 |
| *Pseudomyrmex unicolor* | 1 | 0 | 0 | 1 |
| *Pseudomyrmex urbanus* | 2 | 0 | 0 | 2 |
| *Pseudomyrmex viduus* | 4 | 0 | 0 | 4 |
| *Pyramica beebei* | 0 | 2 | 0 | 2 |
| *Pyramica decipula* | 0 | 15 | 0 | 15 |
| *Pyramica denticulata* | 0 | 87 | 0 | 87 |
| *Pyramica depressiceps* | 0 | 4 | 0 | 4 |
| *Pyramica eggersi* | 4 | 2 | 0 | 6 |
| *Pyramica epinotalis* | 6 | 0 | 0 | 6 |
| *Pyramica glenognatha* | 0 | 1 | 0 | 1 |
| *Pyramica gundlachi* | 0 | 34 | 0 | 34 |
| *Pyramica metopia* | 4 | 6 | 0 | 10 |
| *Pyramica schulzi* | 0 | 1 | 0 | 1 |
| *Pyramica subedentata* | 0 | 2 | 0 | 2 |
| *Pyramica urrhobia* | 0 | 19 | 0 | 19 |
| *Pyramica villiersi* | 0 | 4 | 0 | 4 |
| *Pyramica zeteki* | 0 | 12 | 0 | 12 |
| *Rhopalothrix n. sp.1* | 0 | 1 | 0 | 1 |
| *Rhopalothrix n. sp.2* | 1 | 0 | 0 | 1 |
| *Rhopalothrix n.sp.3* | 2 | 0 | 0 | 2 |
| *Rogeria blanda* | 14 | 2 | 0 | 16 |
| *Rogeria ciliosa* | 0 | 4 | 0 | 4 |
| *Rogeria JSC-001* | 0 | 1 | 0 | 1 |
| *Rogeria JSC-002* | 0 | 1 | 0 | 1 |
| *Rogeria lirata* | 0 | 1 | 0 | 1 |
| *Rogeria micromma* | 0 | 7 | 0 | 7 |
| *Rogeria scobinata* | 0 | 36 | 0 | 36 |
| *Rogeria subarmata* | 7 | 0 | 0 | 7 |
| *Rogeria tonduzi* | 0 | 4 | 0 | 4 |
| *Rogeria unguispina* | 0 | 4 | 0 | 4 |
| *Sericomyrmex sp.1* | 0 | 15 | 0 | 15 |
| *Sericomyrmex sp.2* | 0 | 34 | 0 | 34 |
| *Solenopsis SC-02* | 61 | 2 | 0 | 63 |
| *Solenopsis SC-03* | 30 | 1 | 0 | 31 |
| *Solenopsis SC-05* | 70 | 3 | 4 | 77 |
| *Solenopsis SC-06* | 91 | 41 | 2 | 134 |
| *Solenopsis SC-08* | 2 | 80 | 24 | 106 |
| *Solenopsis SC-09* | 7 | 27 | 4 | 38 |
| *Solenopsis SC-10* | 0 | 0 | 8 | 8 |
| *Solenopsis SC-11* | 1 | 33 | 8 | 42 |
| *Solenopsis SC-12* | 0 | 1 | 6 | 7 |
| *Solenopsis SC-13* | 2 | 0 | 0 | 2 |
| *Solenopsis SC-14* | 0 | 2 | 1 | 3 |
| *Solenopsis SC-15* | 0 | 0 | 4 | 4 |
| *Solenopsis SC-16* | 0 | 4 | 0 | 4 |
| *Solenopsis SC-17* | 0 | 16 | 0 | 16 |
| *Solenopsis virulens* | 0 | 15 | 4 | 19 |
| *Stegomyrmex connectens* | 0 | 1 | 0 | 1 |
| *Stegomyrmex manni* | 0 | 2 | 0 | 2 |
| *Strumigenys cosmostela* | 0 | 2 | 0 | 2 |
| *Strumigenys dolichognatha* | 0 | 3 | 0 | 3 |
| *Strumigenys elongata* | 0 | 7 | 0 | 7 |
| *Strumigenys incuba* | 0 | 1 | 0 | 1 |
| *Strumigenys perparva* | 0 | 15 | 0 | 15 |
| *Strumigenys precava* | 2 | 4 | 0 | 6 |
| *Strumigenys smithii* | 1 | 2 | 0 | 3 |
| *Strumigenys tococae* | 1 | 0 | 0 | 1 |
| *Strumigenys trinidadensis* | 11 | 3 | 0 | 14 |
| *Strumigenys trudifera* | 0 | 40 | 0 | 40 |
| *Strumigenys vilhenai* | 2 | 0 | 0 | 2 |
| *Tapinoma KTRW-001* | 14 | 0 | 0 | 14 |
| *Tapinoma KTRW-002* | 10 | 0 | 0 | 10 |
| *Tapinoma KTRW-003* | 11 | 0 | 0 | 11 |
| *Tapinoma KTRW-004* | 41 | 0 | 0 | 41 |
| *Trachymyrmex cf. bugnioni* | 0 | 23 | 0 | 23 |
| *Trachymyrmex cf. opulentus* | 0 | 1 | 0 | 1 |
| *Trachymyrmex diversus* | 1 | 34 | 0 | 35 |
| *Trachymyrmex farinosus* | 0 | 13 | 0 | 13 |
| *Trachymyrmex ruthae* | 0 | 9 | 0 | 9 |
| *Tranopelta gilva* | 0 | 0 | 19 | 19 |
| *Tranopelta n. sp.* | 0 | 0 | 5 | 5 |
| *Tranopelta subterranea* | 0 | 11 | 13 | 24 |
| *Typhlomyrmex pusillus* | 0 | 4 | 0 | 4 |
| *Typhlomyrmex rogenhoferi* | 0 | 2 | 0 | 2 |
| *Wasmannia auropunctata* | 38 | 108 | 1 | 147 |
| *Wasmannia cf. lutzi* | 2 | 45 | 2 | 49 |
| *Wasmannia iheringi* | 62 | 0 | 0 | 62 |
| *Wasmannia rochai* | 7 | 0 | 0 | 7 |
| *Wasmannia scrobifera* | 17 | 3 | 0 | 20 |
