# Supplementary material for: Species Diversity and Distribution Patterns of the Ants of Amazonian Ecuador
Source: PLoS One. 2010 Oct 1;5(10):e13146. doi: 10.1371/journal.pone.0013146 (PMC2948521; doi:10.1371/journal.pone.0013146)
Supplement: Table S4 — Functional group designation of ants at TBS. (0.09 MB DOC) [file pone.0013146.s004.doc]

**Table S4.** Functional group designation of ants at TBS.

|  |  | **DIET / FORAGING ECOLOGY** | | | | | | | |
| --- | --- | --- | --- | --- | --- | --- | --- | --- | --- |
|  | **HABITAT** | **Group Hunters** | **Solitary Foragers of Live Prey** | **Scavengers** | **Fungus Growers** | **Army Ant Predators** | **Homopteran Tenders** | **Omnivores** | **Unknown** |
| **NESTING ECOLOGY** | **Subterranean / Soil Nesters** | *Acanthostichus[[1]](#endnote-2)* | *Gnamptogenys[[2]](#endnote-3), Stegomyrmex[[3]](#endnote-4), Amblyopone[[4]](#endnote-5), Tranopelta[[5]](#endnote-6), Pachycondyla[[6]](#endnote-7), Dolopomyrmex[[7]](#endnote-8)* | *Carebara[[8]](#endnote-9), Gnamptogenys[[9]](#endnote-10), Pheidole[[10]](#endnote-11)* | *Mycetarotes[[11]](#endnote-12), Mycocepurus[[12]](#endnote-13), Myrmicocrypta[[13]](#endnote-14), Trachymyrmex[[14]](#endnote-15)* | *Labidus[[15]](#endnote-16), Neivamyrmex[[16]](#endnote-17), Nomamyrmex[[17]](#endnote-18)* | *Acropyga[[18]](#endnote-19)* | *Brachymyrmex[[19]](#endnote-20), Octostruma[[20]](#endnote-21), Megalomyrmex[[21]](#endnote-22)* |  |
| **NESTING ECOLOGY** | **Leaf Litter Nesters** |  | *Pyramica[[22]](#endnote-23), Strumigenys[[23]](#endnote-24), Probolomyrmex[[24]](#endnote-25), Discothyrea[[25]](#endnote-26),* | *Rogeria[[26]](#endnote-27)* |  |  |  | *Brachymyrmex[[27]](#endnote-28), Hypoponera[[28]](#endnote-29)* | *Lachnomyrmex[[29]](#endnote-30)* |
| **NESTING ECOLOGY** | **Inquilines** |  | *Centromyrmex[[30]](#endnote-31), Megalomyrmex[[31]](#endnote-32)* |  |  |  |  | *Megalomyrmex[[32]](#endnote-33)* | *Oxyepoecus[[33]](#endnote-34)* |
| **NESTING ECOLOGY** | **Soil + Leaf Litter nesters** |  |  | *Hylomyrma[[34]](#endnote-35)* |  |  |  | *Solenopsis[[35]](#endnote-36)* |  |
| **NESTING ECOLOGY** | **Ground Nesters** | *Leptogenys[[36]](#endnote-37), Pachycondyla[[37]](#endnote-38)* | *Strumigenys[[38]](#endnote-39), Rhopalothrix[[39]](#endnote-40), Megalomyrmex[[40]](#endnote-41), Pachycondyla[[41]](#endnote-42), Gnamptogenys[[42]](#endnote-43), Tranopelta[[43]](#endnote-44), Gigantiops[[44]](#endnote-45), Cylindromyrmex[[45]](#endnote-46)* | *Crematogaster[[46]](#endnote-47), Nylanderia[[47]](#endnote-48), Pheidole[[48]](#endnote-49)* | *Apterostigma[[49]](#endnote-50), Cyphomyrmex[[50]](#endnote-51), Sericomyrmex[[51]](#endnote-52)* |  |  | *Ectatomma[[52]](#endnote-53), Gnamptogenys[[53]](#endnote-54), Megalomyrmex[[54]](#endnote-55), Pachycondyla[[55]](#endnote-56), Nylanderia[[56]](#endnote-57), Pheidole[[57]](#endnote-58), Strumigenys[[58]](#endnote-59), Paraponera[[59]](#endnote-60), Wasmannia[[60]](#endnote-61)* | *Carebara[[61]](#endnote-62), Carebarella[[62]](#endnote-63)* |
| **NESTING ECOLOGY** | **Above Ground Nesters** |  | *Pachycondyla[[63]](#endnote-64), Odontomachus[[64]](#endnote-65)* | *Ochetomyrmex[[65]](#endnote-66),* | *Acromyrmex[[66]](#endnote-67)* | *Eciton[[67]](#endnote-68), Labidus[[68]](#endnote-69)* |  | *Pheidole[[69]](#endnote-70)* |  |
| **NESTING ECOLOGY** | **Twig Nesters** | *Leptogenys[[70]](#endnote-71)* | *Pyramica[[71]](#endnote-72), Strumigenys[[72]](#endnote-73)* | *Camponotus[[73]](#endnote-74), Crematogaster[[74]](#endnote-75), Nylanderia[[75]](#endnote-76)* |  |  |  | *Gnamptogenys[[76]](#endnote-77), Megalomyrmex[[77]](#endnote-78), Pheidole[[78]](#endnote-79), Solenopsis[[79]](#endnote-80)* |  |
| **NESTING ECOLOGY** | **Above Ground + Canopy or Foliage Nesters** |  | *Thaumatomyrmex[[80]](#endnote-81)* | *Camponotus[[81]](#endnote-82), Crematogaster[[82]](#endnote-83)* |  |  |  | *Pheidole[[83]](#endnote-84), Camponotus[[84]](#endnote-85), Brachymyrmex[[85]](#endnote-86)* |  |
| **NESTING ECOLOGY** | **Log Nesters** |  | *Pyramica[[86]](#endnote-87), Anochetus[[87]](#endnote-88), Basiceros[[88]](#endnote-89), Typhlomyrmex[[89]](#endnote-90), Pachycondyla[[90]](#endnote-91)* | *Camponotus[[91]](#endnote-92)* |  |  |  | *Crematogaster[[92]](#endnote-93), Pheidole[[93]](#endnote-94)* |  |
| **NESTING ECOLOGY** | **Canopy Nesters** |  | *Pachycondyla[[94]](#endnote-95), Acanthoponera[[95]](#endnote-96), Gnamptogenys[[96]](#endnote-97), Odontomachus[[97]](#endnote-98), Strumigenys[[98]](#endnote-99), Platythyrea[[99]](#endnote-100)* | *Camponotus[[100]](#endnote-101), Crematogaster[[101]](#endnote-102), Dolichoderus[[102]](#endnote-103), Myrmelachista[[103]](#endnote-104), Nylanderia[[104]](#endnote-105), Tapinoma[[105]](#endnote-106), Wasmannia[[106]](#endnote-107)* |  |  | *Pseudomyrmex[[107]](#endnote-108)* | *Azteca[[108]](#endnote-109), Brachymyrmex[[109]](#endnote-110), Camponotus[[110]](#endnote-111), Cephalotes[[111]](#endnote-112), Dolichoderus[[112]](#endnote-113), Gnamptogenys[[113]](#endnote-114), Pachycondyla[[114]](#endnote-115), Pheidole[[115]](#endnote-116), Procryptocerus[[116]](#endnote-117), Pseudomyrmex[[117]](#endnote-118), Solenopsis[[118]](#endnote-119)* | *Nesomyrmex[[119]](#endnote-120)* |
| **NESTING ECOLOGY** | **Ubiquitous Nesters** |  |  |  |  |  |  | *Wasmannia[[120]](#endnote-121)* |  |
| **NESTING ECOLOGY** | **Unknown** |  | *Pachycondyla[[121]](#endnote-122), Prionopelta[[122]](#endnote-123), Anochetus[[123]](#endnote-124)* | *Nylanderia[[124]](#endnote-125)* |  |  |  | *Pachycondyla[[125]](#endnote-126), Pheidole[[126]](#endnote-127)* |  |

1. *Acanthostichus quadratus* (Brown 2000) [↑](#endnote-ref-2)
2. *Gnamptogenys simulans* [↑](#endnote-ref-3)
3. *Stegomyrmex connectens, S. manni* [↑](#endnote-ref-4)
4. *Amblyopone cf. cleae* [↑](#endnote-ref-5)
5. *Tranopelta gilva, T. n. sp.* [↑](#endnote-ref-6)
6. *Pachycondyla harpax* [↑](#endnote-ref-7)
7. *Dolopomyrmex n. sp*. (Cover & Deyrup 2007) [↑](#endnote-ref-8)
8. *Carebara paya* [↑](#endnote-ref-9)
9. *Gnamptogenys haenschi* [↑](#endnote-ref-10)
10. *Pheidole fimbriata* (Mertl 2009) [↑](#endnote-ref-11)
11. *Mycetarotes acutus, M. unknown* (Brown 2000) [↑](#endnote-ref-12)
12. *Mycocepurus smithii* (Brown 2000) [↑](#endnote-ref-13)
13. *Myrmicocrypta cf. longinoda, M. cf. rudiscapa, M. longinoda* (Brown 2000) [↑](#endnote-ref-14)
14. *Trachymyrmex cf. bugnioni, T. cf. opulentus, T. diversus, T. farinosus, T. ruthae* (Brown 2000) [↑](#endnote-ref-15)
15. *Labidus coecus, L. praedator* [↑](#endnote-ref-16)
16. *Neivamyrmex pseudops, N. punctaticeps* (Brown 2000) [↑](#endnote-ref-17)
17. *Nomamyrmex esenbecki* [↑](#endnote-ref-18)
18. *Acropyga decedens, A. donisthorpei, A. fuhrmanni, A. guianensis* (Brown 2000) [↑](#endnote-ref-19)
19. *Brachymyrmex cavernicola, B. KTRW-001* [↑](#endnote-ref-20)
20. *Octostruma iheringi, O. KTRW-002, O. KTRW-003, O. KTRW-004, O. KTRW-005, O. KTRW-006, O. KTRW-007, O. KTRW-008 (Brown 2000)* [↑](#endnote-ref-21)
21. *Megalomyrmex foreli* [↑](#endnote-ref-22)
22. *Pyramica beebei, P. decipula, P. eggersi, P. epinotalis, P. glenognatha, P. gundlachi, P. metopia, P. schulzi, P. subedentata, P. villiersi, P. zeteki* [↑](#endnote-ref-23)
23. *Strumigenys dolichognatha, S. incuba, S. perparva, S. tococae, S. trudifera, S. vilhenai* [↑](#endnote-ref-24)
24. *Probolomyrmex petiolatus* (Brown 2000) [↑](#endnote-ref-25)
25. *Discothyrea denticulata, D. horni, D. JSC-001, D. sexarticulata* [↑](#endnote-ref-26)
26. *Rogeria blanda, R. ciliosa, R. JSC-001, R. JSC-002, R. lirata, R. micromma, R. scobinata, R. subarmata, R. tonduzi* (Brown 2000) [↑](#endnote-ref-27)
27. *Brachymyrmex KTRW-005, B. KTRW-007, B. KTRW-014, B. KTRW-015* [↑](#endnote-ref-28)
28. *Hypoponera c.f. creola, H. c.f. distinguenda, H. c.f. inexorata, H. c.f. parva, H. perplexa, H. STD10, H. STD11, H. STD12, H. STD13, H. STD15, H. STD16, H. STD17, H. STD19, H. STD20, H. STD21, H. STD22* [↑](#endnote-ref-29)
29. *Lachnomyrmex scrobiculatus* (Brown 2000) [↑](#endnote-ref-30)
30. *Centromyrmex alfaroi* (Brown 2000) [↑](#endnote-ref-31)
31. *Megalomyrmex balzani* [↑](#endnote-ref-32)
32. *Megalomyrmex mondabora* (Adams and Longino 2007) [↑](#endnote-ref-33)
33. *Oxyepoecus ephippiatus* (Brown 2000) [↑](#endnote-ref-34)
34. *Hylomyrma blandiens, H. dolichops, H. immanis, H. praepotens, H. sagax* [↑](#endnote-ref-35)
35. *Solenopsis SC-09, S. SC-10, S. SC-11, S. SC-12, S. SC-13, S. SC-14, S. SC-15, S. SC-16, S. SC-17* [↑](#endnote-ref-36)
36. *Leptogenys gaigei, L. imperatrix, L. ritae* [↑](#endnote-ref-37)
37. *Pachycondyla laevigata* [↑](#endnote-ref-38)
38. *Strumigenys elongata, S. trinidadensis* [↑](#endnote-ref-39)
39. *Rhopalothrix n. sp. 1, R. n. sp. 2, R. n. sp. 3* [↑](#endnote-ref-40)
40. *Megalomyrmex cuatiara* [↑](#endnote-ref-41)
41. *Pachycondyla apicalis, P. constricta* [↑](#endnote-ref-42)
42. *Gnamptogenys horni, G. striatula* [↑](#endnote-ref-43)
43. *Tranopelta subterranea* [↑](#endnote-ref-44)
44. *Gigantiops destructor* [↑](#endnote-ref-45)
45. *Cylindromyrmex godmani* [↑](#endnote-ref-46)
46. *Crematogaster flavomicrops, C. levior, C. stollii* [↑](#endnote-ref-47)
47. *Nylanderia KTRW004* [↑](#endnote-ref-48)
48. *Pheidole araneoides* (Wilson 2003) [↑](#endnote-ref-49)
49. *Apterostigma auriculatum, A. sp. 1, A. sp. 2, A. sp. 3, A. sp. 4, A. sp. 5, A. sp. 6, A. sp. 7* (Brown 2000) [↑](#endnote-ref-50)
50. *Cyphomyrmex cf. major sp. 1, C. cf. minutus sp.1, C. cf. minutus sp. 2, C. cf. rimosus, C. costatus, C. laevigatus, C. sp. 2, C. sp. 3, C. vorticis* (Brown 2000) [↑](#endnote-ref-51)
51. *Sericomyrmex sp. 1, S. sp. 2* (Brown 2000) [↑](#endnote-ref-52)
52. *Ectatomma edentatum, E. lugens, E. tuberculatum* (Brown 2000) [↑](#endnote-ref-53)
53. *Gnamptogenys cf. sulcata, G. kempfi, G. mediatrix, G. mina, G. moelleri, G. sulcata* [↑](#endnote-ref-54)
54. *Megalomyrmex n. sp. near drifti, M. silvestrii, M. timbira* [↑](#endnote-ref-55)
55. *Pachycondyla arhuaca, P. impressa* [↑](#endnote-ref-56)
56. *Nylanderia cf. steinheili* [↑](#endnote-ref-57)
57. *Pheidole astur, P. biconstricta, P. deima, P. fracticeps, P. horribilis, P. midas, P. nitella, P. peruviana, P. triplex* (Wilson, 2003) [↑](#endnote-ref-58)
58. *Strumigenys cosmostela* [↑](#endnote-ref-59)
59. *Paraponera clavata* (Brown 2000) [↑](#endnote-ref-60)
60. *Wasmannia cf. lutzi* [↑](#endnote-ref-61)
61. *Carebara angulata, C. panamensis, C. urichi* [↑](#endnote-ref-62)
62. *Carebarella KTRW-001* [↑](#endnote-ref-63)
63. *Pachycondyla verenae* [↑](#endnote-ref-64)
64. *Odontomachus biumbonatus, O. haematodus, O. meinerti, O. yucatecus* [↑](#endnote-ref-65)
65. *Ochetomyrmex neopolitus, O. semipolitus* (Brown 2000) [↑](#endnote-ref-66)
66. *Acromyrmex coronatus* [↑](#endnote-ref-67)
67. *Eciton hamatum, E. vagans* [↑](#endnote-ref-68)
68. *Labidus punctaticeps* [↑](#endnote-ref-69)
69. *Pheidole amazonica, P. cephalica, P. xanthogaster* [↑](#endnote-ref-70)
70. *Leptogenys nigricans n. sp.* [↑](#endnote-ref-71)
71. *Pyramica depressiceps* [↑](#endnote-ref-72)
72. *Strumigenys precava* [↑](#endnote-ref-73)
73. *Camponotus planatus* [↑](#endnote-ref-74)
74. *Crematogaster nigropilosa, C. sotobosque* [↑](#endnote-ref-75)
75. *Nylanderia KTRW003* [↑](#endnote-ref-76)
76. *Gnamptogenys KTRW-001, G. pleurodon* [↑](#endnote-ref-77)
77. *Megalomyrmex incisus* [↑](#endnote-ref-78)
78. *Pheidole ademonia, P. allarmata, P. ALM-022, P. ALM-025, P. ALM-028, P. gagates, P. lemnisca, P. metana, P. pholeops, P. scolioceps* [↑](#endnote-ref-79)
79. *Solenopsis SC-06, S. SC-08* [↑](#endnote-ref-80)
80. *Thaumatomyrmex (Thaumatomyrmex)* (Brown 2000) [↑](#endnote-ref-81)
81. *Camponotus arboreus, C. bradleyi, C. branneri, C. cacicus, C. callistus, C. claviscapus, C. emeryodicatus, C. eurynotus, C. helleri, C. integellus, C. latangulus, C. macrochaeta, C. mocsaryi, C. mus, C. nidulans, C. orthocephalus, C. sexguttatus, C. WM-001, C. WM-002, C. WM-003, C. WM-004, C. WM-005, C. WM-006, C. WM-010, C. wytsmani* [↑](#endnote-ref-82)
82. *Crematogaster carinata* [↑](#endnote-ref-83)
83. *Pheidole ALM-023, P. cramptoni, P. laidlowi, P. sagax, P. tobini* [↑](#endnote-ref-84)
84. *Camponotus bispinosus, C. rapax* [↑](#endnote-ref-85)
85. *Brachymyrmex KTRW-004, B. KTRW-006, B. KTRW-008, B. KTRW-010, B. KTRW-011, B. KTRW-013, B. KTRW-016, B. KTRW-017, B. KTRW-018* [↑](#endnote-ref-86)
86. *Pyramica denticulata* [↑](#endnote-ref-87)
87. *Anochetus diegensis, A. mayri* (Brown 2000) [↑](#endnote-ref-88)
88. *Basiceros conjugans, B. manni, B. militaris* (Wilson and Hoelldobler 1986) [↑](#endnote-ref-89)
89. *Typhlomyrmex pusillus, T. rogenhoferi* (Brown 2000) [↑](#endnote-ref-90)
90. *Pachycondyla obscuricornis* [↑](#endnote-ref-91)
91. *Camponotus formiciformis* [↑](#endnote-ref-92)
92. *Crematogaster limata* [↑](#endnote-ref-93)
93. *Pheidole ALM-031*  [↑](#endnote-ref-94)
94. *Pachycondyla carinulata, P. crenata, P. unidentata* [↑](#endnote-ref-95)
95. *Acanthoponera minor, A. peruviana* (Brown 2000) [↑](#endnote-ref-96)
96. *Gnamptogenys regularis* [↑](#endnote-ref-97)
97. *Odontomachus hastatus, O. mayi, O. panamensis* [↑](#endnote-ref-98)
98. *Strumigenys smithii* [↑](#endnote-ref-99)
99. *Platythyrea angusta* (Brown 2000) [↑](#endnote-ref-100)
100. *Camponotus abscisus, C. bidens, C. brevis, C. constructor, C. excisus, C. nitidior, C. novogranadensis, C. senex, C. WM-007, C. WM-008, C. WM-009* [↑](#endnote-ref-101)
101. *Crematogaster acuta, C. brasiliensis, C. crucis, C. curvispinosa, C. egregior, C. erecta, C. foliocrypta, C. JTL-022, C. JTL-026, C. rochai, C. tenuicula* [↑](#endnote-ref-102)
102. *C. attelaboides, D. bidens, D. decollatus, D. imitator, D. inpai, D. lamellosus, D. lobicornis, D. lutosus, D. quadridenticulatus, D. shattucki, D. validus, D. varians* [↑](#endnote-ref-103)
103. *Myrmelachista KTRW-001, M. KTRW-002, M. KTRW-003, M. KTRW-004, M. KTRW-005, M. KTRW-006, M. KTRW-007, M. KTRW-008, M. KTRW-009* [↑](#endnote-ref-104)
104. *Nylanderia KTRW001, P. KTRW002, P. KTRW006* [↑](#endnote-ref-105)
105. *Tapinoma KTRW-001, T. KTRW-002, T. KTRW-003, T. KTRW-004* (Brown 2000) [↑](#endnote-ref-106)
106. *Wasmannia iheringi, W. rochai, W. scrobifera* [↑](#endnote-ref-107)
107. *Pseudomyrmex viduus* [↑](#endnote-ref-108)
108. *Azteca SJ-A, A. SJ-AA, A. SJ-B, A. SJ-BB, A. SJ-C, A. SJ-CC, A. SJ-D, A. SJ-DD, A. SJ-E, A. SJ-F, A. SJ-FF, A. SJ-G, A. SJ-GG, A. SJ-H, A. SJ-HH, A. SJ-I, A. SJ-II, A. SJ-J, A. SJ-JJ, A. SJ-KK, A. SJ-LL, A. SJ-M, A. SJ-MM, A. SJ-N, A. SJ-NN, A. SJ-O, A. SJ-OO, A. SJ-P, A. SJ-PP, A. SJ-Q, A. SJ-R* (Brown 2000) [↑](#endnote-ref-109)
109. *Brachymyrmex KTRW-002, B. KTRW-003, B. KTRW-009, B. KTRW-012, B. KTRW-019* [↑](#endnote-ref-110)
110. *Camponotus atriceps, C. femoratus, C. linnaei* [↑](#endnote-ref-111)
111. *Cephalotes atratus, C. cordatus, C. laminatus, C. maculatus, C. manni, C. marginatus, C. minutus, C. n. sp. near maculatus, C. n. sp. near palta, C. pallidus, C. pavonii, C. peruviensis, C. ramiphilus, C. spinosus, C. umbraculatus* (Andrade and Baroni Urbani 1999) [↑](#endnote-ref-112)
112. *Dolichoderus diversus, D. laminatus* [↑](#endnote-ref-113)
113. *Gnamptogenys concinna, G. n. sp. A, G. n. sp. B* [↑](#endnote-ref-114)
114. *Pachycondyla aenescens, P. cavinodis, P. foetida, P. globosa, P. inversa, P. rostrata, P. striatinodis, P. villosa* [↑](#endnote-ref-115)
115. *Pheidole ALM-033, P. floricola, P. gilva, P. pubiventris* [↑](#endnote-ref-116)
116. *Procryptocerus attenuatus, P. coriarius, P. hylaeus, P. impressus, P. n. sp. near eladio, P. nalini, P. paleatus, P. pictipes* (Longino and Snelling 2002) [↑](#endnote-ref-117)
117. *Pseudomyrmex atripes, P. colei, P. duckei, P. eduardi, P. elongatus, P. ethicus, P. faber, P. filiformis, P. gracilis, P. oculatus, P. pupa, P. rochai, P. sericeus, P. simplex, P. sp. nr.cladoicus, P. sp. nr.maculatus, P. sp. nr.spiculus, P. sp. PSW-161, P. sp. PSW-37, P. sp. PSW-52, P. sp. PSW-58, P. sp. PSW-59, P. spiculus, P. subater, P. tenuis, P. terminalis, P. unicolor, P. urbanus* (Brown 2000) [↑](#endnote-ref-118)
118. *Solenopsis SC-02, S. SC-03, S. SC-05* [↑](#endnote-ref-119)
119. *Nesomyrmex argentinus, N. brasiliensis, N. costatus, N. echinatinodis, N. pleuriticus, N. rutilans, N. spininodis, N. tristani* (Kempf 1959) [↑](#endnote-ref-120)
120. *Wasmannia auropunctata* [↑](#endnote-ref-121)
121. *Pachycondyla marginata* [↑](#endnote-ref-122)
122. *Prionopelta amabilis* (Brown 2000) [↑](#endnote-ref-123)
123. *Anochetus bispinosus* [↑](#endnote-ref-124)
124. *Nylanderia 9, P. cf. fulva, P. KTRW005, P. KTRW008* [↑](#endnote-ref-125)
125. *Pachycondyla lunaris, P. crassinoda, P.gilberti, P.oberthueri* [↑](#endnote-ref-126)
126. *Pheidole ALM-006, P.ALM-013, P.ALM-026, P.ALM-032, P.ALM-034, P.exigua, P.sabella, P.sarpedon* (Mertl 2009) [↑](#endnote-ref-127)
